# Efficient Removal of Carcinogenic Azo Dyes from Water Using Iron(II) Clathrochelate Derived Metalorganic Copolymers Made from a Copper-Catalyzed [4 + 2] Cyclobenzannulation Reaction

**DOI:** 10.3390/polym15132948

**Published:** 2023-07-04

**Authors:** Noorullah Baig, Suchetha Shetty, Rupa Bargakshatriya, Sumit Kumar Pramanik, Bassam Alameddine

**Affiliations:** 1 Department of Mathematics and Natural Sciences, Gulf University for Science and Technology, Mubarak Al-Abdullah 32093, Kuwait; 2 Functional Materials Group, Gulf University for Science and Technology, Mubarak Al-Abdullah 32093, Kuwait; 3 CSIR-Central Salt and Marine Chemicals Research Institute, Gijubhai Badheka Marg, Bhavnagar 364002, Gujarat, Indiasumitpramanik@csmcri.res.in (S.K.P.)

**Keywords:** metalorganic copolymer, [4 + 2] cycloaddition, cyclobenzannulation, dye removal, environmental remediation

## Abstract

A novel synthetic strategy is disclosed to prepare a new class of metalorganic copolymers that contain iron(II) clathrochelate building blocks by employing a mild and cost-effective copper-catalyzed [4 + 2] cyclobenzannulation reaction, using three specially designed diethynyl iron(II) clathrochelate synthons. The target copolymers CBP1-3 were isolated in high purity and excellent yields as proven by their structural and photophysical characterization, namely, Fourier transform infrared (FTIR), X-ray photoelectron spectroscopy (XPS) and UV–VIS absorption and emission spectroscopies. The thermogravimetric analysis (TGA) of CBP1-3 revealed an excellent chemical stability. Investigation of the adsorption properties of the target copolymers towards the carcinogenic methyl red dye from aqueous solution revealed a quantitative uptake in 30 min. Isothermal adsorption studies disclosed that methyl red uptake from aqueous solution followed the Langmuir model for all of the target copolymers, reaching a maximum adsorption capacity (q_m_) of 431 mg g^−^. Kinetic investigation revealed that the adsorption followed pseudo-first-order with an equilibrium adsorption capacity (q_e,cal_) of 79.35 mg g^−^ and whose sorption property was sustained even after its reuse several times.

## 1. Introduction

Dyes are predominantly utilized as coloring agents in the textile industry and are employed in a myriad of products, such as pharmaceuticals, food and beverage, leather, plastics, cosmetics, and paper [1,2]. Dyes, whose global annual production is estimated to 7 × 10^7^ tons [2,3], present several advantages, namely, the variety of their color palette, ease of application on various types of materials, structural diversity, and low energy consumption [4,5]. There are several methods to classify synthetic dyes, notably that are based on the chemical structure of their chromophore, hence, they could be grouped as acidic, basic, azoic, nitro, sulphur, etc. [6,7]. It is noteworthy that azo dyes, which are characterized by the presence of one or more azo groups (-N=N-), are widely employed in industry, with a production rate that exceeds half that of the total dyes synthesized annually [8,9]. Despite its undeniable importance and contribution to economic development [5,10], the textile industry is one of the largest global polluters as it consumes large amounts of fuels and chemical reagents [11,12,13]. Additionally, the textile industry uses massive quantities of freshwater in the various operations required for its production chain, such as washing, bleaching, and dyeing [14], thus producing effluents that contain high concentrations of pollutants [15]. Inappropriate discharge practices of azo dye waste lead to harmful environmental effects and severely damage aquatic life [16,17]. On top of this, azo dyes cause health complications as they are considered carcinogenic, mutagenic, and teratogenic [18,19,20], while their high solubility in water allows them to access the food chain, consequently causing many health problems, mainly fever, renal damage, and cramps [5]. Methyl red (MR) is an anionic mono-azo dye (c.f. Table S1) that is used in paper printing and textile dyeing. Nevertheless, MR is classified as carcinogenic and mutagenic, besides being considered as an eye and skin irritant and it causes damage to the aquatic life if present in excess [19,21]. 

Various technologies have been tested to remove azo dyes from wastewater, ranging from physicochemical methods, such as oxidation and irradiation, to electrochemical techniques, such as coagulation–flocculation, photodegradation, ozone treatment, electro Fentons, and hypochlorite usage [22,23,24] These technologies suffer from several disadvantages, among others, the high dosage required and the production of large amounts of sludge, which make them economically unattractive [10]. Nevertheless, the use of adsorbents has been proven to be promising due to its high efficiency, cost effectiveness, simplicity, and possibility to be used under ambient conditions [5].

Metal clathrochelates derivatives, which have been known for more than five decades, consist of three-dimensional octahedral inorganic complexes with an encapsulated central metal ion [25,26,27]. Among these metalorganic complexes, a myriad of iron(II) clathrochelate derivatives doubly capped with aryl borate groups were synthesized, disclosing several advantages, notably, their versatile synthesis from environmentally friendly synthons, modular chemistry allowing for their functionalization, high chemical stability, intricate structure, and cost effectiveness [28,29,30,31]. Interestingly, iron(II) clathrochelates revealed remarkable properties for applications in biosensors, catalysts for hydrogen generation, materials for electronic transport, organogels, and building blocks to make supramolecular cages and metalorganic frameworks (MOFs) [32,33,34,35,36,37,38]. Several adsorbents derived from iron(II) clathrochelate were made and tested against various gases and dyes, disclosing remarkable properties [39,40,41,42]. This work discloses the synthesis of three new metalorganic copolymers, CBP1-3, in a high yield and under mild reaction conditions using a copper-catalyzed [4 + 2] cyclobenzannulation reaction of specially designed diethynyl iron(II) clathrochelate synthons CM1-3, which were made in one step, with 2,5-bis(phenylethynyl)terephthalaldehyde 6. The resulting metalorganic copolymers CBP1-3 were investigated as adsorbents of the carcinogenic Methyl Red dye from aqueous solution, revealing the excellent adsorption of the latter.

## 2. Materials and Methods

All of the reactions were carried out under an inert atmosphere of dry argon. Unless otherwise specified, the chemical reagents were used without further purification, as purchased from Merck (Darmstadt, Germany), Aldrich (Darmstadt, Germany), Alfa Aesar, Honeywell (New Hampshire, United States), Loba Chemie (Mumbai, India), and HiMedia (Mumbai, India). Decane-5,6-dione dioxime 1, 2-((4-(tert-butyl)phenyl)ethynyl)benzaldehyde 5, and 2,5-bis(phenylethynyl)terephthalaldehyde 6 were synthesized following the literature [34,43]. Synthon 4 was synthesized following a reported procedure [44,45] and its structure was confirmed by ^1^H- and ^13^C- nuclear magnetic resonance (NMR) and electrospray ionization mass spectrometry (ESI-MS) (c.f. synthetic procedure (i) and Figures S1, S6 and S11). Anhydrous solvents, namely, hexane, tetrahydrofuran (THF), dichloroethane (DCE), dichloromethane (DCM), chloroform (CHCl_3_) methanol, and diisopropylamine (^i^Pr_2_NH), were further dried over molecular sieves and deoxygenated by bubbling with dry argon gas for 30 min. Thin layer chromatography (TLC) was performed on aluminum sheets coated with silica gel 60 F254 and revealed using a UV lamp. NMR spectra (^1^H: 600 MHz, ^13^C: 150 MHz) were recorded using a JEOL resonance ECZ600R spectrometer at 25 °C, using CDCl_3_ and CD_2_Cl_2_ as a solvent with the chemical shifts (δ) given in ppm and referenced to tetramethylsilane (TMS). Electro-spray ionization mass spectra (ESI-MS) were recorded on waters QTOF Micro YA263 using a single quadrapole detector-2 (SQD-2). CHNS analysis was performed by weighing the samples on a Sartorius ultra-micro balance with ±0.1 µg resolution. Sample weights ranging from 1.0 and 2.0 mg were used. Calibration was done using standard reference material: 2, 5-bis-(5-tert-butyl-2- benzoxazol-2-yl)-thiophenone, C_26_H_26_N_2_O_2_S. CHNS analysis was performed on an Elementar CHNSO analyzer manufactured by Eurovector. The instrument uses flash combustion and analyses the product gases by gas chromatography online. Signals were detected using a Thermal Conductivity Detector supplied by the manufacturer. O-analysis used the HT 1400 high temperature unit coupled to the above instrument. Carbon monoxide was used as analytical species to quantify oxygen. UV–VIS spectra were recorded using a Shimadzu UV1800 spectrophotometer. FT-IR spectra were recorded on a PerkinElmer G spectrophotometer using a KBr matrix. Thermogravimetric analysis (TGA) was recorded on a Mettler Toledo Star SW 8.10 system (model number TGA/SDTA851e) analyzer and it was used to measure the thermal stability of the composites from room temperature to 800 °C with a heating rate of 10 °C/min under an inert atmosphere. X-ray photoelectron spectroscopy (XPS) data were recorded with a Thermo scientific using a monochromatic Al K-radiation source (1486.6 eV) with a spot size of samples of 10 mm × 10 mm × 5 mm. Spectra acquisition and processing were carried out using the software Thermo Advantage Version 4.87. The base pressure in the XPS analysis chamber was in the range of 10^−10^ to 10^−9^ torr. The analyzer was operated with a pass energy of 20 eV, dwell time of 50 min and with a step size of 0.1 eV. Brunauer–Emmett–Teller (BET) surface area were performed at 77 K using a liquid nitrogen bath (77 K) on a Quantachrome Quadrasorb automatic volumetric instrument. All of the samples were outgassed for 12 h at 120 °C under vacuum prior to the gas adsorption studies. The surface areas were evaluated using the Brunauer–Emmett–Teller (BET) model applied between P/P_0_ values of 0.05 and 0.3 for the samples. The pore size distributions were calculated using the non-localized density functional theory (NLDFT) method. The surface area of each of the sample was measured multiple times and then averaged out for a proper comparison. 

### 2.1. Synthesis

#### 2.1.1. Synthesis of CM1 (Procedure A)

2-(4-((4-(tert-butyl)phenyl)ethynyl)phenyl)-4,4,5,5-tetramethyl-1,3,2-dioxaborolane 4 (540 mg, 1.5 mmol, 2.3 eq.), decane-5,6-dione dioxime 1 (405 mg, 2.0 mmol, 3 eq.), and iron(II) chloride (FeCl_2_, 83 mg 0.66 mmol, 1 eq.) in 6 mL of degassed methanol were refluxed under argon for 24 h. The solvent was evaporated under reduced pressure and the orange red solid was precipitated from methanol. The resulting orange red precipitate was filtered and washed with methanol and hexane, followed by diethyl ether. Yield: (691 mg, 93 %). ^1^H-NMR (CDCl_3_, 600 MHz, ppm): *δ* 7.70 (d, *J* = 7.7 Hz, 4H, ArH), 7.55 (d, *J* = 8.0 Hz, 4H, ArH), 7.48 (d, *J* = 8.3 Hz, 4H, ArH), 7.38 (d, *J* = 8.1 Hz, 4H, ArH), 2.83 (t, *J* = 7.7 Hz, 12H, N=C-CH_2_), 1.58 (m, 12H, CH_2_), 1.36–1.33 (m, 30H, CH_2_ & CH_3_), 0.90 (t, 18H, CH_3_); ^13^C-NMR (CDCl_3_, 150 MHz, ppm): *δ* 157.14 (C=N), 151.24, 131.68, 131.38, 130.59, 125.37, 122.39, 120.86, 89.91, 88.91, 34.84, 31.29, 29.28, 27.19, 22.52, 13.93; ESI-MS; Calcd. for M^•+^ C_66_H_88_B_2_FeN_6_O_6_: 1138.63; found 1138.63 FTIR (KBr, cm^−1^): 2958 (Aliphatic C-H str), 2211 (CΞC str), 1468 (Aliphatic C-H ben) 1396 (B-O str), 1184 (B-C str), 828 (Ar-C-H ben).

#### 2.1.2. Synthesis of CM2

CM2 was prepared following procedure A, with 2-(4-((4-(tert-butyl)phenyl)ethynyl)phenyl)-4,4,5,5-tetramethyl-1,3,2-dioxaborolane 4 (280 mg, 0.77 mmol, 2.3 eq.), Cyclohexane-1,2-dione dioxime 2 (147 mg, 1.0 mmol, 3 eq.), and iron(II) chloride (FeCl_2_, 43 mg 0.34 mmol, 1 eq.) in 5 mL of degassed methanol. Brick-red solid (298 mg, 91%). ^1^H-NMR (600 MHz, CD_2_Cl_2_, ppm): *δ* 7.69 (*d*, *J* = 8.1 Hz, 6H, ArH), 7.53–7.51 (*brm*, 6H, ArH), 7.45 (*d, J* = 8.4 Hz, 4H, ArH), 2.97 (*brs*, 12H, CH_2_), 1.86 (*brs*, 12H, CH_2_), 1.38 (*brs*, 18H, CH_3_); ^13^C-NMR (150 MHz, CD_2_Cl_2_, ppm): δ 152.66 (C=N), 142.14, 135.11, 132.25, 131.74, 130.93, 126.00, 123.02, 121.00, 90.00, 89.48, 35.26, 31.48, 26.80, 22.17; ESI-MS; Calcd. for M^•+^ C_54_H_58_B_2_FeN_6_O_6_: 964.39; found 964.39 FTIR (KBr, cm^−1^): 2947 (Aliphatic C-H str), 2208 (CΞC str), 1431 (Aliphatic C-H ben) 1399 (B-O str), 1194 (B-C str), 829 (Ar-C-H ben).

#### 2.1.3. Synthesis of CM3

CM3 was prepared following procedure A, with 2-(4-((4-(tert-butyl)phenyl)ethynyl)phenyl)-4,4,5,5-tetramethyl-1,3,2-dioxaborolane 4 (270 mg, 0.75 mmol, 2.3 eq.), *anti*-diphenylglyoxime 3 (243 mg, 1.0 mmol, 3 eq.), and iron(II) chloride (FeCl_2_, 42 mg 0.33 mmol, 1 eq.) in 5 mL of degassed methanol. Brick-red solid (382 mg, 94%). ^1^H-NMR (600 MHz, CDCl_3_, ppm): δ 7.77 (d, J = 8.0 Hz, 4H, ArH), 7.51 (d, J = 8.0 Hz, 4H, ArH), 7.46 (m, 14H, ArH), 7.37 (d, J = 8.3 Hz, 6H, ArH), 7.32–7.28 (m, 18H, ArH), 1.35 (brs, 18H, CH_3_); ^13^C-NMR (150 MHz, CDCl_3_, ppm): δ 155.89 (C=N), 151.76, 134.65, 131.81, 131.47, 131.02, 130.81, 130.27, 129.84, 127.74, 126.31, 125.44, 120.21, 90.98, 89.00, 34.88, 31.25; CHN analysis: (Found C, 74.4; H, 5.10; N, 6.66 Calcd. for C_78_H_64_B_2_FeN_6_O_6_ C, 74.42; H, 5.12; N, 6.68%), FTIR (KBr, cm^−1^): 2973 (Aliphatic C-H str), 2203 (CΞC str), 1450 (Aliphatic C-H ben) 1396 (B-O str), 1144 (B-C str), 868 (Ar-C-H ben).

#### 2.1.4. Synthesis of CBM

A Schlenk tube was charged under argon with 2-((4-(tert-butyl)phenyl)ethynyl) benzaldehyde 5 (27 mg, 0.10 mmol, 2.1 eq.), CM2 (48 mg, 0.05 mmol, 1 eq.), copper(II) triflate Cu(OTf)_2_ (3.6 mg, 0.01 mmol, 0.2 eq.), and trifluoroacetic acid TFA (15 μL, 0.2 mmol) in 4 mL of deoxygenated dichloroethane. The solution was heated at 100 °C overnight and the solvent was evaporated under reduced pressure. The resulting mixture was dissolved in DCM and extracted with a saturated solution of NaHCO_3_ (50 mL × 2). The combined organic layer was washed with deionized water (100 mL × 3), concentrated, and the desired product was isolated using silica gel column chromatography with DCM/hexane (50:50 *v*/*v*) as the eluent. Brick-red solid (55 mg, 95%). ^1^H-NMR (600 MHz, CDCl_3_, ppm): δ 7.81–7.78 (brm, 4H, ArH), 7.57 (t, J = 7.9 Hz, 2H, ArH), 7.48 (d, J = 7.9 Hz, 2H, ArH) 7.43–7.39 (m, 8H, ArH), 7.32–7.26 (m, 4H, ArH), 7.20–7.11 (m, 8H, ArH), 2.84 (brs, 12H, CH_2_), 1.73 (brs, 12H, CH_2_), 1.25 (*brs*, 18H, CH_3_); ^13^C-NMR (150 MHz, CDCl_3_, ppm): δ 152.48, 150.17, 139.47, 133.19, 132.25, 131.83, 131.74, 130.93, 130.25, 130.10, 129.51, 128.19, 126.64, 126.00, 125.74, 125.58, 125.40, 125.05, 34.39, 31.68, 26.79, 22.17. ESI-MS; Calcd. for M^•+^ C_70_H_70_B_2_FeN_6_O_6_: 1168.48; found 1168.48 FTIR (KBr, cm^−1^): 2947 (Aliphatic C-H str), 1435 (Aliphatic C-H ben), 1391 (B-O str), 1194 (B-C str), 833 (Ar-C-H ben).

#### 2.1.5. Synthesis of Copolymer CBP1 (Procedure B)

2,5-bis(phenylethynyl) terephthalaldehyde 6 (73 mg, 0.22 mmol, 1 eq.), CM1 (250 mg, 0.22 mmol, 1 eq.), Cu(OTf)_2_ (32 mg, 0.09 mmol, 0.4 eq.), and TFA (136 μL, 1.76 mmol) were refluxed in 35 mL of deoxygenated dichloroethane in a Schlenk tube under argon. After 2 days of reaction, the precipitate was filtered and washed exhaustively with a sequence of the following solvents 50 mL of DCM, 50 mL of THF, 50 mL of acetone, 50 mL of water, and 50 mL of diethyl ether, affording a brown solid (235 mg, 83%). FTIR (KBr, cm^−1^): 2955 (Aliphatic C-H str), 1461 (Aliphatic C-H ben), 1396 (B-O str), 1180 (B-C str), 820 (Ar-C-H ben). UV–VIS: (THF, 10^−6^ M), λ_max_ [nm] = 313, 452, fluorescence: (THF, 10^−6^ M), λ_max_ [nm] = 440.

#### 2.1.6. Synthesis of CBP2

CBP2 was prepared following procedure B, with 2,5-bis(phenylethynyl) terephthalaldehyde 6 (63 mg, 0.19 mmol, 1 eq.), CM2 (183 mg, 0.19 mmol, 1 eq.), Cu(OTf)_2_ (27 mg, 0.08 mmol, 0.4 eq.), and TFA (117 μL, 1.52 mmol) in 30 mL of degassed dichloroethane. Brown solid (200 mg, 95%); FTIR (KBr, cm^−1^): 2948 (Aliphatic C-H str), 1450 (Aliphatic C-H ben), 1392 (B-O str), 1190 (B-C str), 830 (Ar-C-H ben). UV–VIS: (THF, 10^−6^ M), λ_max_ [nm] = 312, 450, fluorescence: (THF, 10^−6^ M), λ_max_ [nm] = 440.

#### 2.1.7. Synthesis of CBP3

CBP3 was prepared following procedure B, with: 2,5-bis(phenylethynyl) terephthalaldehyde 6 (60 mg, 0.18 mmol, 1 eq.), CM3 (226 mg, 0.18 mmol, 1 eq.), Cu(OTf)_2_ (26 mg, 0.07 mmol, 0.4 eq.), and TFA (110 μL, 1.44 mmol) in 30 mL of degassed dichloroethane. Brown solid (228 mg, 90%); FTIR (KBr, cm^−1^): 2965 (Aliphatic C-H str), 1449 (Aliphatic C-H ben), 1370 (B-O str), 1200 (B-C str), 890 (Ar-C-H ben); UV–VIS: (THF, 10^−6^ M), λ_max_ [nm] = 297, 481, fluorescence: (THF, 10^−6^ M), λ_max_ [nm] = 451.

## 3. Results and Discussion

### 3.1. Synthesis 

**Scheme 1:**
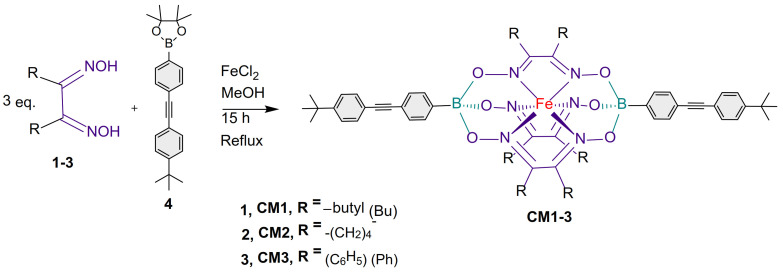
Synthesis of comonomers CM1-3.

Scheme 1 discloses the reaction conditions to synthesize comonomers CM1-3 from a one-pot complexation reaction [31,34] of iron(II) chloride (FeCl_2_, 0.44 eq.) with a dioxime derivative 1-3 (1.35 eq.) in refluxing methanol overnight and in the presence of the diboronic acid pinacol ester capping reagent 4 (1.0 eq.), thus affording the desired building blocks in excellent yields. The structures of the desired comonomers CM1-3 were confirmed by ^1^H- and ^13^C-nuclear magnetic resonance (NMR), electrospray ionization mass spectra (ESI-MS), CHN analysis, and FTIR spectroscopy (Figures S2–S4, S7–S9, S12 and S13 and Table S2). 

#### 3.1.1. Synthesis of the Prototypical Monomer CBM

**Scheme 2:**
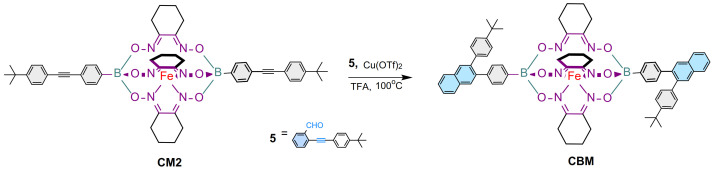
Synthesis of CBM.

**Figure 1:**
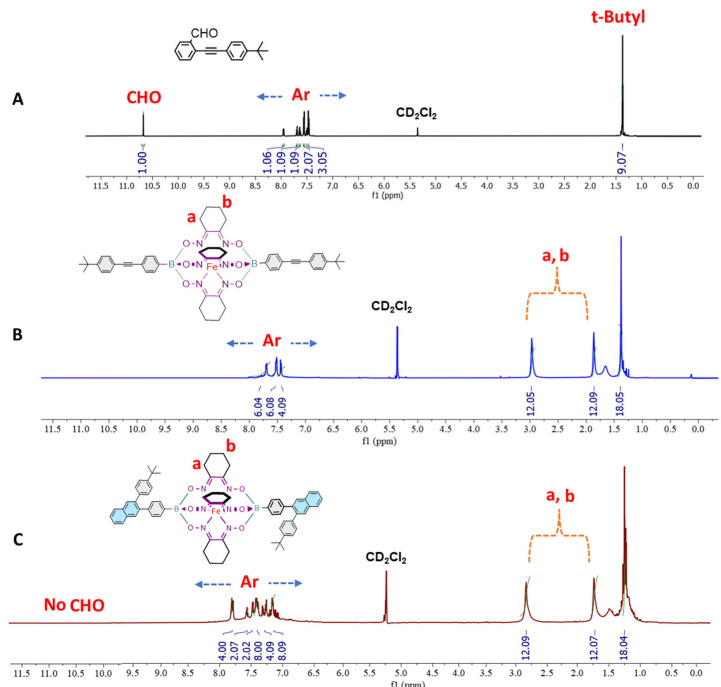
Comparative ^1^H-NMR spectra of 5 (**A**), CM2 (**B**), and CBM (**C**). Labels a and b: characteristic methylene (-CH_2_-) peaks of the peripheral cyclohexyl groups.

As a proof of concept, the prototypical iron(II) clathrochelate monomer CBM was prepared using the copper-catalyzed [4 + 2] cyclobenzannulation reaction conditions, where the diethynyl-containing iron(II) clathrochelate synthon CM2 was reacted with two equivalents of 2-((4-(tert-butyl)phenyl)ethynyl) benzaldehyde 5 in the presence of copper(II) triflate and trifluoracetic acid (TFA) in refluxing dichloroethane overnight, affording CBM in a quantitative yield (Scheme 2). The structure of the latter was confirmed by ^1^H- and ^13^C-nuclear magnetic resonance (NMR), ESI-MS, and FTIR spectroscopy (Figure 1 and Figures S5, S10, S14 and S18).

Figure 1 portrays the comparative ^1^H-NMR spectra of the starting materials 5 and CM2 with CBM, where the spectrum of the latter clearly confirmed the presence of all of the desired peaks and the disappearance of those that could be attributed to synthon 5, namely, the fingerprint chemical shifts of the carbaldehyde group at 10.66 ppm and the t-butyl group at 1.37 ppm. The chemical shifts in the ^1^H-NMR spectrum of CBM ranging from 7.1 ppm to 7.8 ppm are assigned to the aromatic protons, while those detected at 2.8 ppm and 1.73 ppm are attributed to the characteristic methylene (-CH_2_-) protons of the cyclohexyl groups (c.f. peaks labeled a, b in Figure 1). Likewise, the chemical shifts observed at 1.25 ppm are assigned to the methyl (-CH_3_) protons of the tertiary butyl group (Figure 1). ^13^C-NMR spectrum of CBM displays all of the expected aromatic peaks in the range of 152.4–125.0 ppm in addition to the chemical shifts of the aliphatic carbons of both the cyclohexyl and tertiary butyl groups at 34.9 ppm, 31.6 ppm, 26.7 ppm, and 22.1 ppm (Figure S10). In addition, the high purity of CBM was confirmed by electrospray ionization mass spectrometry (ESI-MS, Figure S14). 

#### 3.1.2. Synthesis of Copolymers CBP1-3

**Scheme 3:**
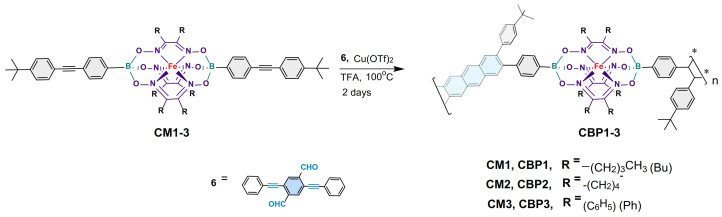
Synthesis of copolymers CBP1-3.

Synthesis of the target copolymers CBP1-3 (Scheme 3) was carried out using reaction conditions similar to those employed to make the prototypical monomer CBM described in Scheme 2. The copper-catalyzed [4 + 2] cyclobenzannulation reaction of the diethynyl iron(II) clathrochelate derivatives CM1-3 and 2,5-bis(phenylethynyl)terephthalaldehyde 6 afforded the target copolymers CBP1-3 in excellent yields in the range of 83–95% (Scheme 3). Table 1 summarizes the attempts carried out to optimize the copolymerization reaction conditions: when a 2.5 × 10^−2^ M solution of 2,5-bis(phenylethynyl)terephthalaldehyde 6 and an equimolar amount of CM1 are reacted in refluxing dichloroethane (DCE) in the presence of Cu(OTf)_2_ and TFA for 48 h, and CBP1 was isolated as an insoluble solid in 48% yield (Table 1, entry 1). Thus, to improve the reaction yield, the concentration of comonomers CM1 and 6 was diluted to a molar concentration of 1.25 × 10^−2^ M, which afforded CBP1 in 65% (Table 1, entry 2). Further dilution of the comonomers to a concentration of 6 × 10^−3^ M resulted in the improvement of the reaction yield, affording CBP1 in 83% (Table 1, entry 3).

Similar reaction conditions were employed in the copolymerization of 6 in the presence of an equimolar amount of either CM2 or CM3, thus affording CBP2 and CBP3 in 95% and 90% yields, respectively (Table 1, entry 4 and 5).

**Figure 2:**
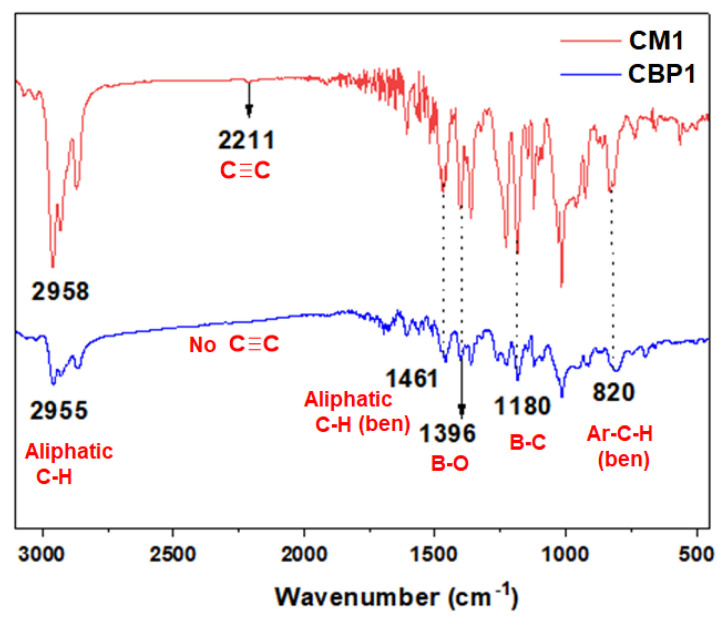
Comparative FT-IR spectrum of CM1 (red) and CBP1 (blue).

**Figure 3:**
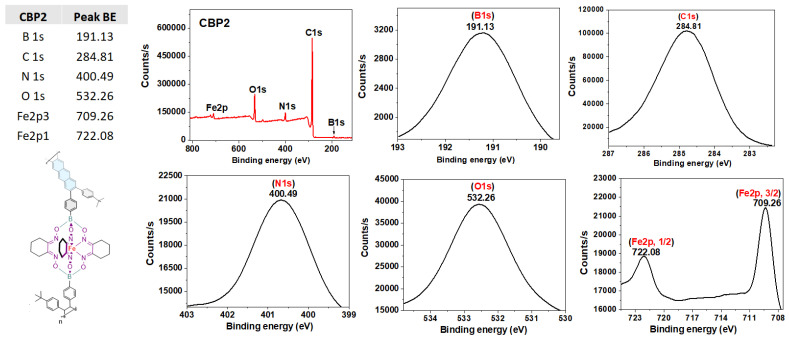
High-resolution XPS survey scan and spectra of B1s, C1s, N1s, O1s, and Fe2p of copolymer **CBP2**.

**Figure 4:**
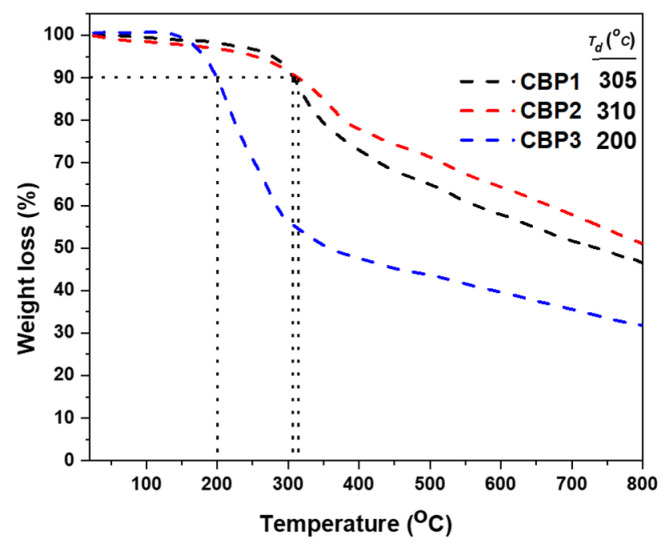
TGA thermograms of polymers CBP1-3, T*_d_* represents the temperature for 10% weight loss.

**Figure 5:**
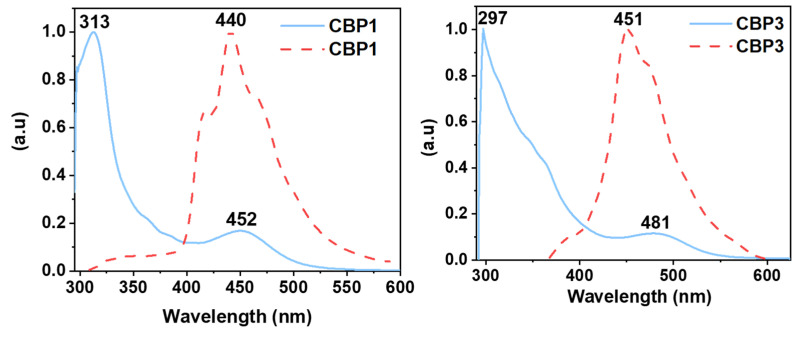
Normalized UV–VIS absorption (solid lines) and emission (dotted lines) spectra of **CBP1-3** (CM = 10^−8^ M in THF).

CBP1-3 was characterization by various techniques, namely, FTIR, XPS, TGA, UV–VIS absorption, and emission spectroscopies (Figure 2, Figure 3, Figure 4 and Figure 5 and Figures S19–S21, S22, S23 and S25). Nevertheless, the target copolymers were found to be insoluble in common organic solvents, such as THF, DCM, DMSO, methanol, acetone, and chloroform, which prevented their molar mass determination.

Figure 2 portrays the comparative FTIR absorption spectra for comonomer CM1 and its corresponding target copolymer CBP1. The characteristic stretching vibrations of the ethynyl (C≡C) group were detected at ~2211 cm^−1^ [46] for CM1, which disappeared from the spectrum of copolymer CBP1. It is noteworthy that the absorption bands identified at ~2955 cm^−1^ and ~1461 cm^−1^ correspond to the distinctive aliphatic C-H stretching and bending vibrations, respectively, which clearly indicates the presence of the butyl groups in CBP1 [47,48]. In addition, the fingerprint stretching vibrations were confirmed for each of the B-O (1396 cm^−1^), B-C (1180 cm^−1^), and aromatic C-H (820 cm^−1^) bending vibration peaks, which further supports the formation of target copolymer CBP1 [34,49]. Likewise, the FTIR absorption spectra of target copolymers CBP2,3 revealed their distinctive stretching and bending vibration peaks, which corroborated their successful formation (Figure 2 and Figures S19–S21).

X-ray photoelectron spectroscopy (XPS) survey-scan spectra of CBP1-3 confirm the presence of all of their constituting elements. The C1s, O1s, and N1s binding energies were detected in the range of ~284.8–284.7 eV, 532.2 eV, and 400.5-400.1 eV, respectively, whereas those for B1s and Fe2p were revealed in the range of 191.1–191.0 eV and 708.9-722.1 eV, respectively (Figure 3 and Figures S22 and S23) [34]. 

Figure 4 illustrates the thermogravimetric analysis (TGA) of copolymers CBP1-3 depicting their 10% weight loss temperatures in the range of 200–310 °C, which indicates their relatively high thermal stability.

Interestingly, the UV–VIS absorption and emission spectra of the target copolymers CBP1-3 displayed similar features. The butyl- and cyclohexyl-containing iron(II) clathrochelate cyclobenzannulated copolymers CBP1,2 revealed a similar UV absorption band at ~312, whereas the one with phenyl side groups, i.e., CBP3, displayed a strong absorption band at 297 (Figure 5). The emission spectra of CBP1,2 portrayed a broad peak with an intensity maximum at 442 nm, while the phenyl-containing copolymer CBP3 disclosed an emission maximum at 451 nm (Figure 5 and Figure S25).

The porosity of the target copolymers CBP1-3 was investigated using nitrogen adsorption–desorption experiments at 77 K and low relative pressure (Figures S26–S28). The Brunauer–Emmett–Teller (BET) method revealed a surface area of 74.0 m^2^g^−1^ for the cyclohexyl-containing copolymer CBP2, whereas those with butyl- and phenyl- side groups, i.e., CBP1 and CBP3, showed lower BET surface areas of 7.0 m^2^g^−1^ and 35.0 m^2^g^−1^, respectively. The pore volumes of CBP1-3 derived from these isotherms disclosed values of 0.014 cm^3^ g^−1^, 0.081 cm^3^ g^−1^, and 0.030 cm^3^ g^−1^, respectively.

### 3.2. Methyl Red Adsorption Studies

**Figure 6:**
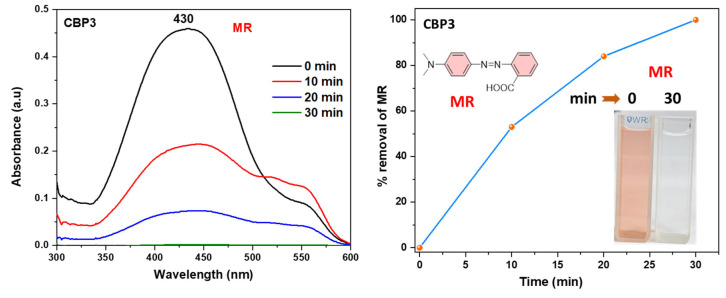
UV–VIS absorption spectra of the aqueous solution of MR in the presence of CBP3 at various time intervals (left) and plot of % removal of MR dye versus time (right, inset: chemical structure of MR and photographs showing the color change upon dye adsorption).

Copolymers CBP1-3 were tested as adsorbents of the cancerogenic and mutagenic azo dye methyl red (MR) purchased from Merck^®^ (CAS 493527). The uptake capacity was evaluated by soaking an aliquot of CBP1-3 in an aqueous solution of MR (Figure 6 and Figures S29 and S30). The removal efficiency of MR by copolymers CBP1-3 was investigated by recording the UV–VIS absorbance spectra of the dye’s aqueous solutions at different time intervals. The dye adsorption experiments were carried out by stirring a 5 mg sample of a given copolymer in a 5 mL aqueous solution of MR (20 mg L^−1^, pH = 7) at an ambient temperature. The adsorption efficiency, E (%), and amount of dye adsorbed by the copolymer, q_e_ (mg g^−1^), were calculated using the following equations [25]:

E (%) = (C_0_ − C_e_)/C_0_ × 100

q_e_ (mg g^−1^) = (C_0_ − C_e_) V/m

where C_0_ and C_e_ are the initial and equilibrium dye concentrations (mg L^−1^), respectively; m (g) is the quantity of the adsorbent used; and V (L) is the volume of dye solution.

The absorbance maximum peak intensity of MR detected at 430 nm noticeably decreased upon the addition of the target polymers CBP1-3 to the solution, which confirmed the latter to be very good adsorbents. Interestingly, all of the copolymers reached 100% adsorption capacity of MR, but at different time intervals, with CBP3 being the fastest by quantitatively removing MR from the aqueous solution in 30 min at room temperature (Figure 6 and Figures S29 and S30). It is worthwhile to note that the experiments were run three times and the adsorption values were reproducible.

To better comprehend the adsorption behavior of copolymers CBP1-3, the adsorption isotherms of the MR removal experiments were obtained by preparing different aqueous solutions of MR with initial concentrations ranging from 50 to 600 mg L^−1^, where Langmuir and Freundlich linear isotherm models were employed to fit the adsorption isotherm data. In the case of the Langmuir isotherm model, the following linear equation was utilized [25]:

1/q_e_ = 1/K_L_q_m_ × 1/C_e_ + 1/q_m_

On the other hand, the linear equation below was employed for the Freundlich isotherm model [25]:

Log q_e_ = Log K_F_ + 1/n Log C_e_

where q_e_ (mg g^−1^) denotes the equilibrium adsorption capacity, C_e_ (mg L^−1^) represents the equilibrium dye concentration, and q_m_ (mg g^−1^) indicates the maximum adsorption capacity. K_L_ is the Langmuir constant, whereas K_F_ and n are Freundlich constants correlated to the sorption capacity and sorption intensity, respectively (Figures S31–S33). The Langmuir parameters were obtained by plotting the graph of 1/q_e_ versus 1/C_e_, and those for Freundlich were derived from the plot of log q_e_ versus log C_e_ (Figures S31–S33). Both models were used to fit the equilibrium data obtained for the MR adsorption. It is worthwhile to note that the correlation coefficient (R^2^) derived from the linear equation using the Langmuir model was found to be higher than that computed for the Freundlich isotherm model of MR (Figure S33), thus implying that the Langmuir isotherm is a more favorable model to illustrate the equilibrium data, and which suggests a homogenous adsorption and the formation of monolayers of MR dye on the adsorbates CBP1-3. Additionally, the maximum adsorption capacity (q_m_) derived from the Langmuir model was found to be 199.20 mg g^−1^ and 219.8 mg g^−1^ for CBP2 and CBP1, respectively, and it reached 431.03 mg g^−1^ for the phenyl-bearing iron(II) clathrochelate cyclobenzannulated copolymer CBP3, which, to the best of our knowledge, was superior to the adsorption capacity values for most of the materials reported in the literature [5,24,50].

**Figure 7:**
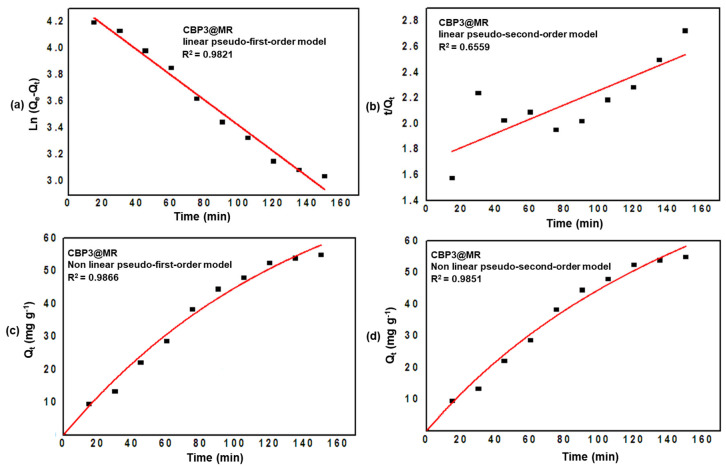
Kinetic modelling of MR by CBP3 using linear pseudo-first-order (**a**) second-order (**b**) and nonlinear pseudo-first-order s(**c**) and second-order (**d**) kinetic models.

Linear and nonlinear pseudo-first- and pseudo-second-order kinetic experiments were carried out to better understand the adsorption mechanism of MR on CBP3 using an initial concentration of 500 mg L^−1^ of MR dye at different time intervals every 15 min, up to 150 min (Figure 7).

The linear equation that is detailed below was employed to investigate the pseudo-first-order model [34]:

ln (q_e_ − q_t_) = ln q_e_ − k_1_t

Alternatively, the linear pseudo-second-order model is expressed by the following [34]:

t/q_t_ = t/q_e_ + 1/k_2_q_e_^2^

Additionally, in order to circumvent possible erroneous correlations by the linear equations above, nonlinear correlations were also employed to check the pseudo-first-order and the pseudo-second-order models using the respective equations [51,52]:

q_t_ = q_e_(1 − e^−k^_1_^t^)

and

q_t_ = (q_e_^2^k_2_t)/(1 + q_e_k_2_t)

where q_e_ (mg g^−1^) and q_t_ (mg g^−1^) are the adsorption capacities at equilibrium and time t (min), respectively. k_1_ is the rate constant of the pseudo-first-order model, whereas k_2_ is the rate constant of the pseudo-second-order model.

As shown in Table 2, the calculated adsorption capacity at equilibrium, q_e,cal_, was derived from the linear pseudo-first-order model by plotting ln(q_e_ – q_t_) versus t, whereas it was extrapolated from the plot of t/q_t_ versus t for the linear pseudo-second-order model. Likewise, q_e,cal_ was computed from the nonlinear pseudo-first-order and pseudo-second-order models by plotting q_t_ versus t and applying the relevant equations given above. Interestingly, Table 2 discloses the correlation coefficients, R^2^, of 0.9821 and 0.9866 derived from the respective linear and nonlinear equations of the pseudo-first-order models, which are higher than the ones derived from the linear and nonlinear pseudo-second-order models, R^2^, of 0.6559 and 0.9851, respectively. Moreover, the comparison of the experimental capacity at equilibrium, q_e,exp_ = 76 mg g^−1^ with those calculated, q_e,cal_, of 79.35 mg g^−1^ and 88.43 mg g^−1^ for the linear and nonlinear pseudo-first-order models [53], respectively, clearly reveal a better agreement than those derived from the pseudo-second-order models, thus suggesting that the adsorption of MR by CBP3 follows a pseudo-first-order kinetic model (c.f. the plausible interaction between the copolymers and MR dye in Figure S34).

**Figure 8:**
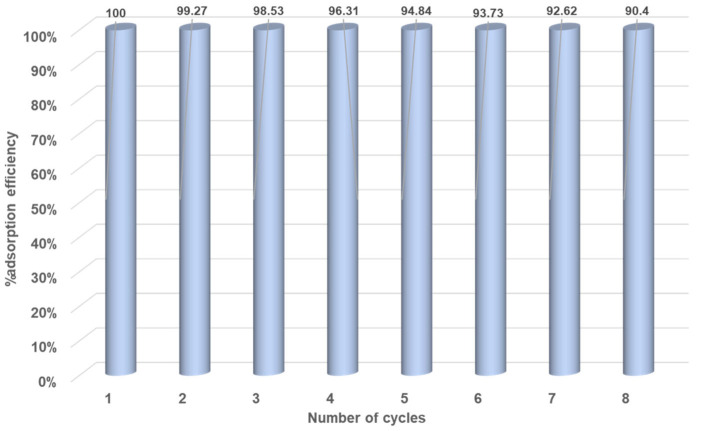
Graphical representation of the MR adsorption recyclability of CBP3.

Reusability experiments were carried out in order to test the adsorbing performance of copolymer CBP3 towards MR after several adsorption–desorption cycles. Thus, a sample of CBP3 copolymer loaded with MR was ultrasonicated in deionized water for 10 min followed by its isolation through vacuum filtration over a membrane filter before adding the regenerated copolymer sample to a freshly prepared aqueous solution of MR. This procedure was repeated for several cycles, proving a removal efficiency of 90.4% for CBP3 even after eight cycles (Figure 8).

## 4. Conclusions

We report the synthesis of a new class of three metalorganic copolymers CBP1-3 bearing iron(II) clathrochelate unit and interlinked by anthracene groups via a typical copper-catalyzed [4 + 2] cycloaddition polymerization reaction conditions. The target copolymers were isolated in excellent yields and revealed excellent removal capacities of the carcinogenic dye methyl red from aqueous medium, especially CBP3, which disclosed an ultrafast and superior adsorption efficiency up to 100% in 30 min and exhibited a maximum adsorption capacity (q_m_) of 431 mg g^−1^ with the possibility to regenerate the hitherto mentioned polymer for several cycles. The novel iron (II) clathrochelate-based copolymers presented herein confer several advantages, particularly a versatile synthesis methodology, low cost, superior stability, and excellent adsorption capacity. Therefore, these materials are prominent candidates for environmental remediation applications, specifically as adsorbents of the hazardous azo dyes.

## Figures and Tables

**Table 1:** Summary of the optimized reaction conditions of copolymers **CBP1-3**.

Entry	Copolymer ^a^	Time in Days	C_M_^b^ [M]	Yield (%)
1	CBP1	2	2.5 × 10^−2^	48
2	CBP1	2	1.25 × 10^−2^	65
3	CBP1	2	6.0 × 10^−3^	83
4	CBP2	2	6.0 × 10^−3^	95
5	CBP3	2	6.0 × 10^−3^	90

^a^: Cu(OTf)_2_ (0.4 eq.), TFA (8.0 eq.), and DCE;^b^ Molar concentration of **6.**

**Table 2:** **Linear and nonlinear** pseudo-first-order and pseudo-second-order kinetic model parameters for the adsorption of MR on CBP3.

Dye on CBP3				Pseudo-First-Order Model			Pseudo-Second-Order Model		
		C_0_ (mg L^−1^)	q_e,exp_ (mg g^−1^)	q_e,cal_ (mg g^−1^)	k_1_ (min^−1^)	R^2^	q_e,cal_ (mg g^−1^)	k_2_ (min^−1^)	R^2^
MR	Linear	500	76	79.35	−0.00013	0.9821	179.21	1.83 × 10^−5^	0.6559
	Non linear			88.43	0.00709	0.9866	148	2.91 × 10^−5^	0.9851

## Data Availability

The raw/processed data required to reproduce these findings can be shared upon demand.

## Supplementary Materials

The following supporting information can be downloaded at: https://www.mdpi.com/article/10.3390/polym15132948/s1, Figures S1–S5: ^1^H-NMR spectra of 4, CM1-3, & CBM; Figures S6–S10: ^13^C-NMR spectra of 4, CM1-3, & CBM; Figures S11–S14: ESI-MS of 4, CM1,2, & CBM; Figures S15–S21: FT-IR of CM1-3, CBM, & CBP1-3; Figures S22 and S23: XPS spectra of copolymers CBP1,3; Figures S24 and S25: UV-VIS absorption spectra of CM1-3 & CBP2; Figures S26–S28: Nitrogen isotherms of CBP1-3; Figures S29 and S30: Methylene red (MR) dye adsorption by CBP1-2; Figures S31–S33: Langmuir & Freundlich isotherm models of MR on CBP1-3; Figure S34: Plausible interaction between the copolymer and MR dye; Table S1: Chemical structures of some azo dyes; Table S2: CHN analysis of CM3.
