# Supplementary material for: Efficient Removal of Carcinogenic Azo Dyes from Water Using Iron(II) Clathrochelate Derived Metalorganic Copolymers Made from a Copper-Catalyzed [4 + 2] Cyclobenzannulation Reaction
Source: Polymers (Basel). 2023 Jul 4;15(13):2948. doi: 10.3390/polym15132948 (PMC10346286; doi:10.3390/polym15132948)
Supplement: Supplementary file 1 [file polymers-15-02948-s001.zip › polymers-2428781-SI.pdf]

Supplementary Materials

Efficient Removal of Carcinogenic Azo Dyes from Water Using Iron(II) Clathrochelate Derived Metalorganic Copolymers Made from a Copper-Catalyzed [4 + 2] Cyclobenzannulation Reaction

Noorullah Baig [1,2], Suchetha Shetty [1,2], Rupa Bargakshatriya [3], Sumit Kumar Pramanik [3] and Bassam Alameddine [1,2,*]

[1] Department of Mathematics and Natural Sciences, Gulf University for Science and Technology, Mubarak Al-Abdullah 32093, Kuwait
[2] Functional Materials Group, Gulf University for Science and Technology, Mubarak Al-Abdullah 32093, Kuwait
[3] CSIR-Central Salt and Marine Chemicals Research Institute, Gijubhai Badheka Marg, Bhavnagar 364002, Gujarat, India; sumitpramanik@csmcri.res.in (S.K.P.)
* Correspondence: alameddine.b@gust.edu.kw; Tel.: +965-2530-7111

Contents

Chemical structures of some azo dyes	Table S1
Synthesis of **4**	Scheme S1
^{1}H-NMR spectra of **4, CM1-3,** & **CBM**	Figure S1-S5
^{13}C-NMR spectra of **4, CM1-3,** & **CBM**	Figure S6-S10
ESI-MS of **4, CM1,2,** & **CBM**	FigureS11-S14
CHN analysis of **CM3**	Table S2
FT-IR of **CM1-3, CBM,** & **CBP1-3**	Figure S15-S21
XPS spectra of copolymers **CBP1,3**	Figure S22-S23
UV-VIS absorption spectra of **CM1-3** & **CBP2**	Figure S24-S25
Nitrogen isotherms of **CBP1-3**	Figure 26-S28
Methylene red (MR) dye adsorption by **CBP1-2**	Figure S29-S30
Langmuir & Freundlich isotherm models of MR on **CBP1-3**	Figure S31-S33
Plausible interaction between the copolymer and MR dye	Figure S34

Table S1: Chemical Structures of some azo dyes

Entry	Dye	Structure
1	Methyl orange	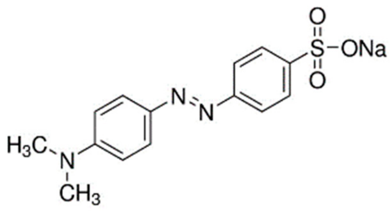
2	Acid yellow 36	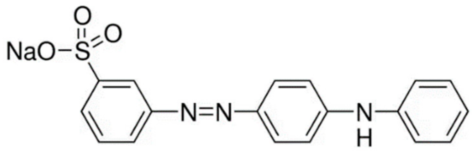
3	Methyl red	
4	Acid Orange 7	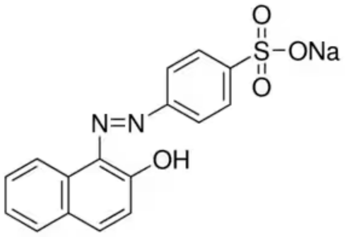
5	Acid Red 18	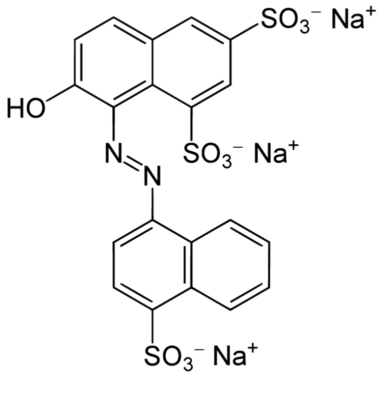

i). Synthesis of 4:

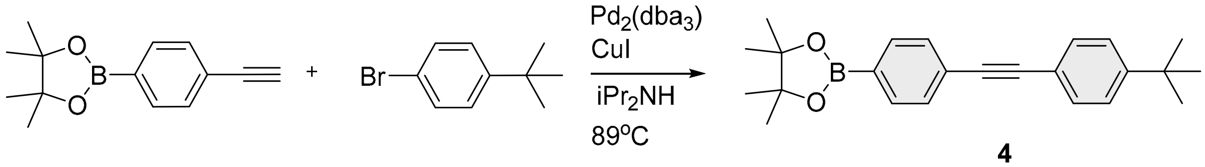

Scheme S1: Synthesis of **4**

A Schlenk tube was charged under argon with 4-Ethynylphenylboronic acid pinacol ester (798 mg, 3.5 mmol, 1 eq.), 1-Bromo-4-tert-butylbenzene (637 mL, 3.6 mmol, 1.05 eq.), $Pd_2(dba)_3$ (19 mg, 2.1 µmol, 6 mol%), PPh_3 (11 mg, 4.2 µmol), and CuI (8 mg, 4.2 µmol) in 8 mL of degassed diisopropylamine (iPr_2NH) and the solution was refluxed for 2 days and the solvent was then evaporated under reduced pressure. The resulting residue was extracted with DCM from an aqueous solution of $NaHCO_3$ and the organic layer was washed with a brine solution followed by deionized water (500 mL × 2). The desired product was isolated by silica gel column chromatography, using DCM/hexane (10:90 v/v) as the eluent. Brown solid (1.2 g, 95%). ^{1}H-NMR (600 MHz, $CDCl_3$, ppm): δ 7.78 (d, 2H, J = 8.0 Hz, ArH), 7.52 (d, 2H, J = 7.7 Hz, ArH), 7.45 (d, 2H, J = 8.3, Hz, ArH) 7.37 (d, 2H, J = 8.3, Hz, ArH) 1.34 (s, 9H, $-CH_3$),1.29 (s, 12H, -CH3). ^{13}C-NMR (150 MHz, $CDCl_3$, ppm): δ 151.76, 134.65, 131.47, 130.81, 127.28, 126.31, 125.44, 120.22, 90.99, 89.01, 84.01, 34.88, 31.25, 34.96. ESI-MS: m/z calculated for M+1 $C_{24}H_{30}BO_2$ 361.23 found 361.21.

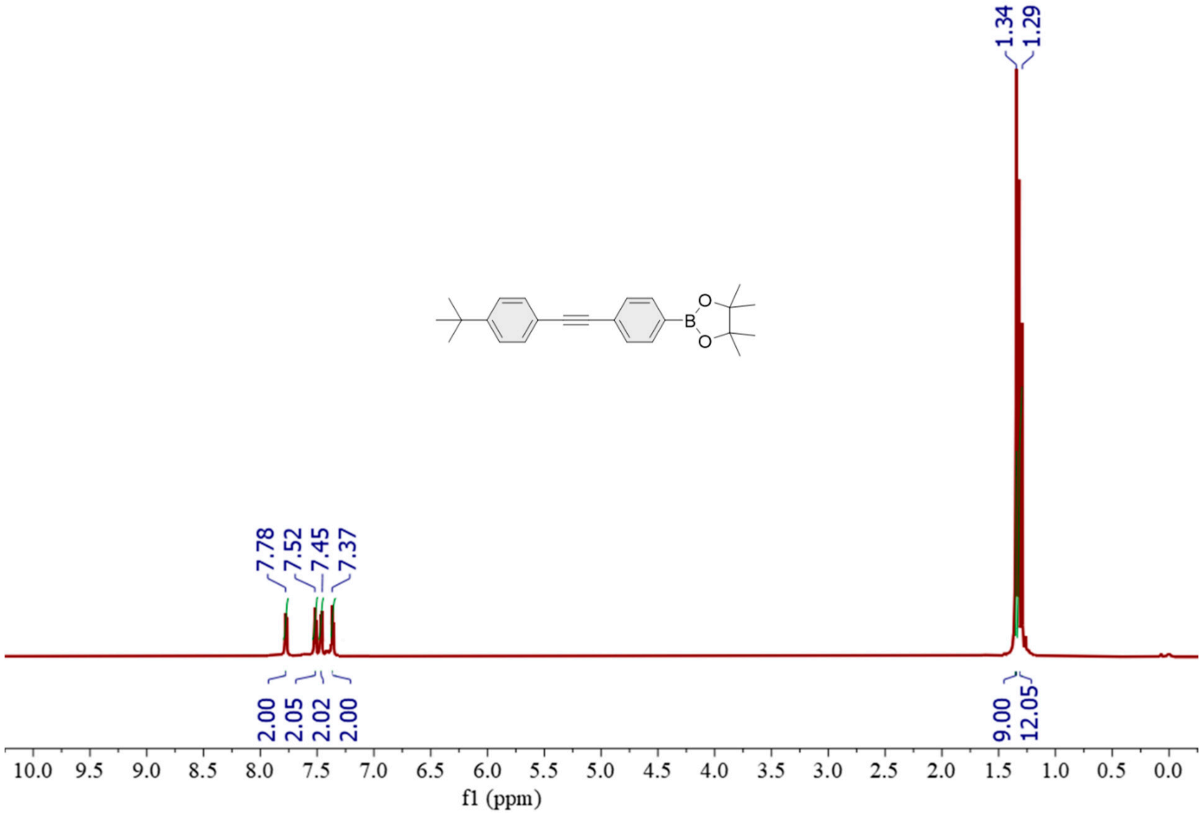

Figure S1: ^{1}H NMR spectrum of **4** ($CDCl_3$, 600 MHz)

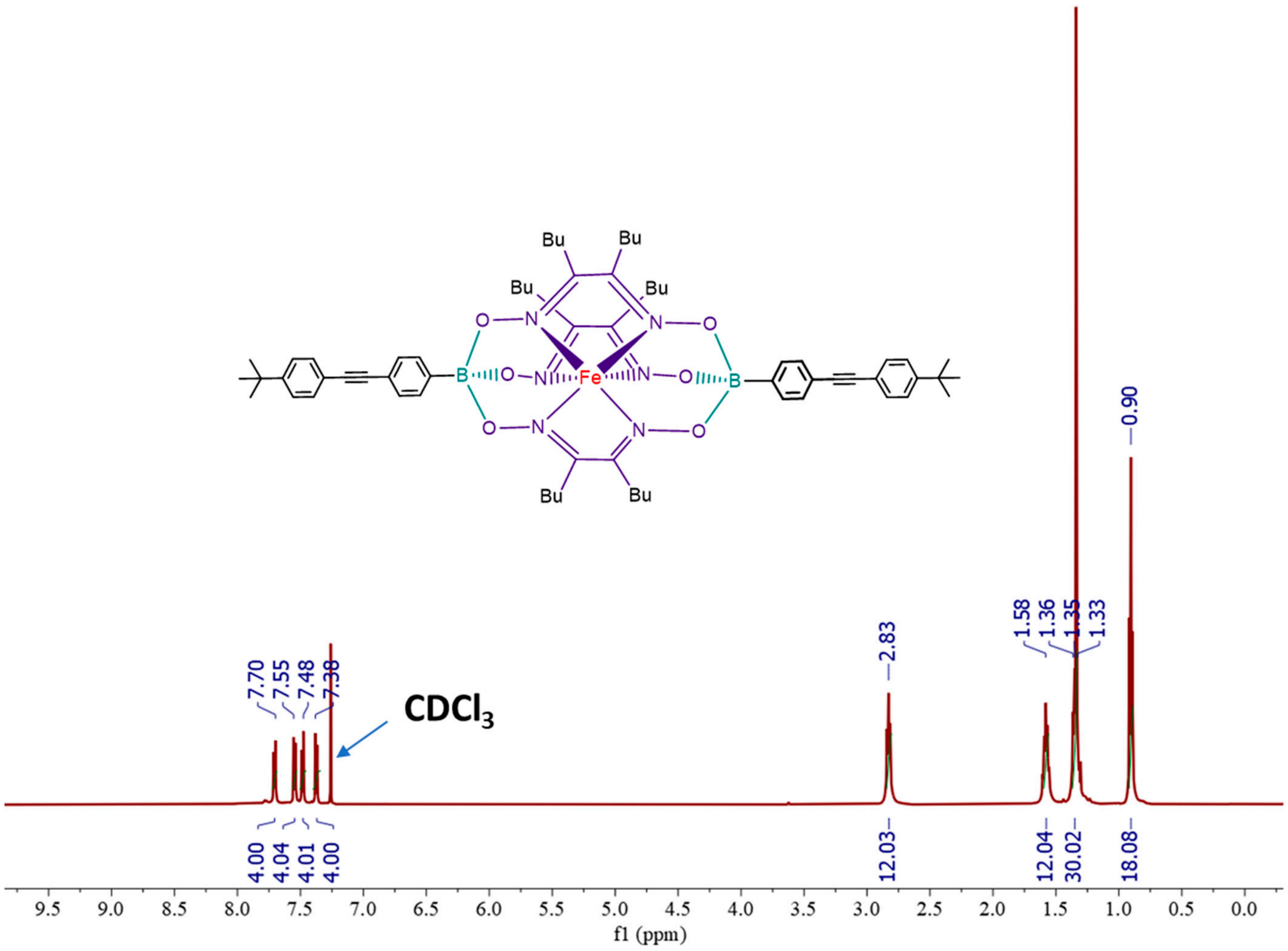

Figure S2: ^{1}H NMR spectrum of **CM1** ($CDCl_3$, 600 MHz)

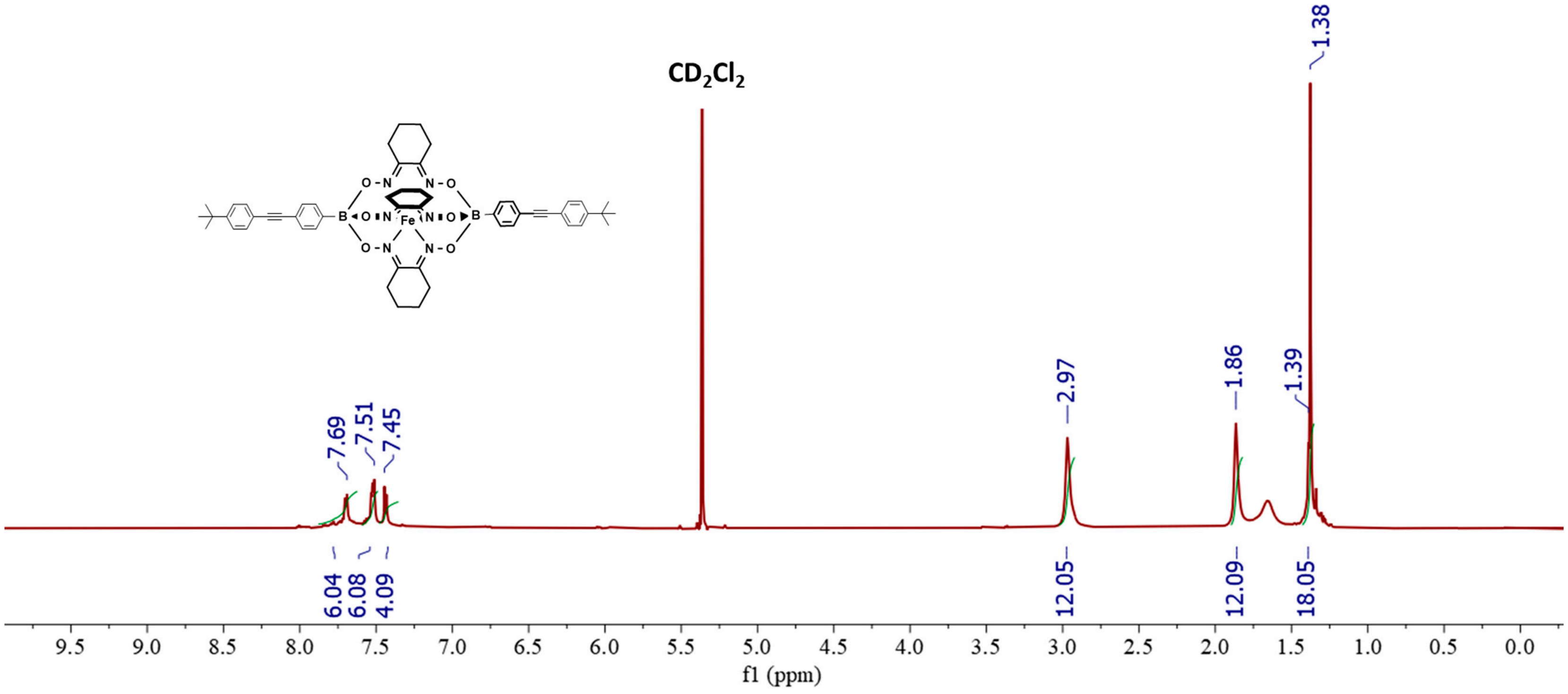

Figure S3: ^{1}H NMR spectrum of **CM2** (CD_2Cl_2, 600 MHz)

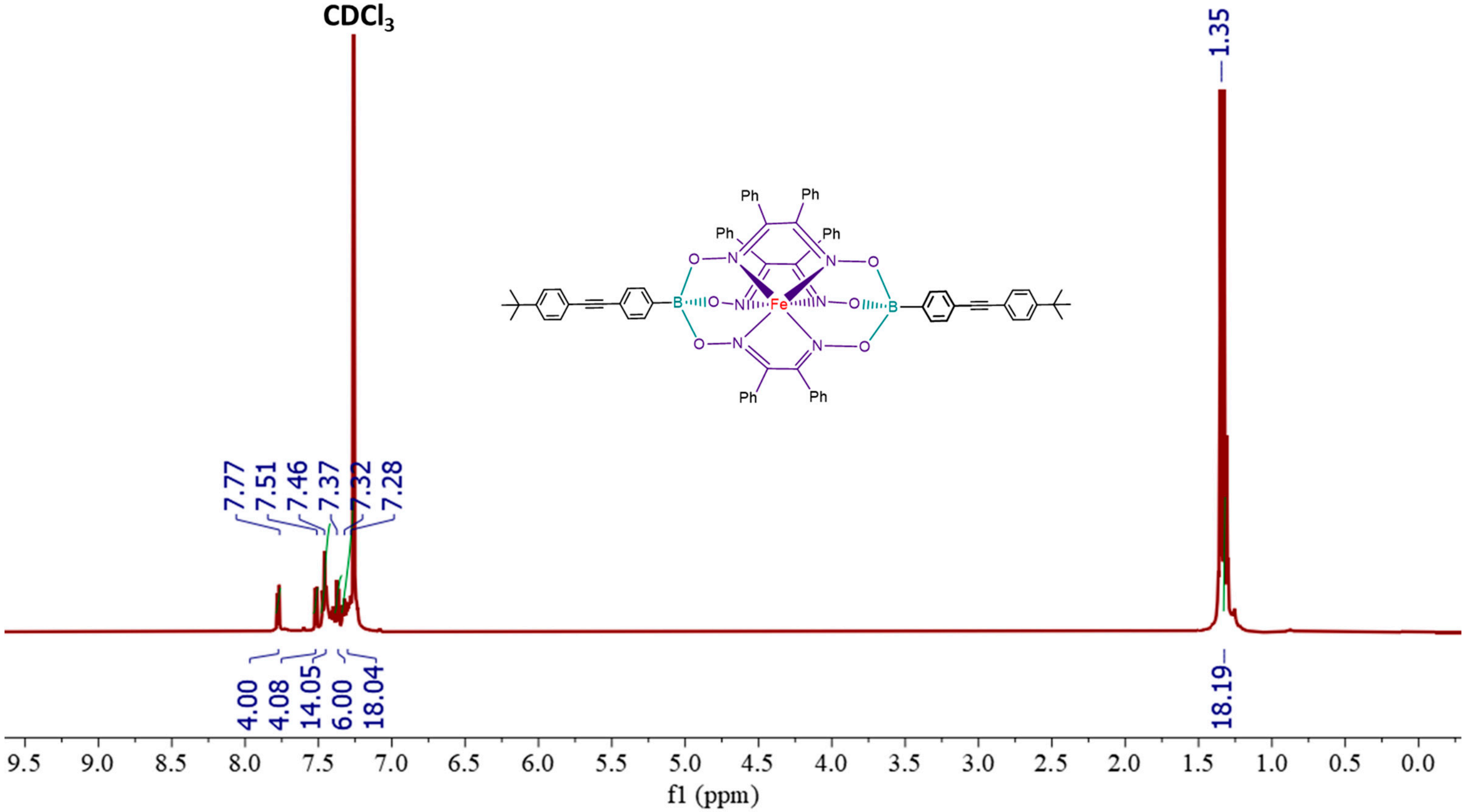

Figure S4: ^{1}H NMR spectrum of **CM3** ($CDCl_3$, 600 MHz)

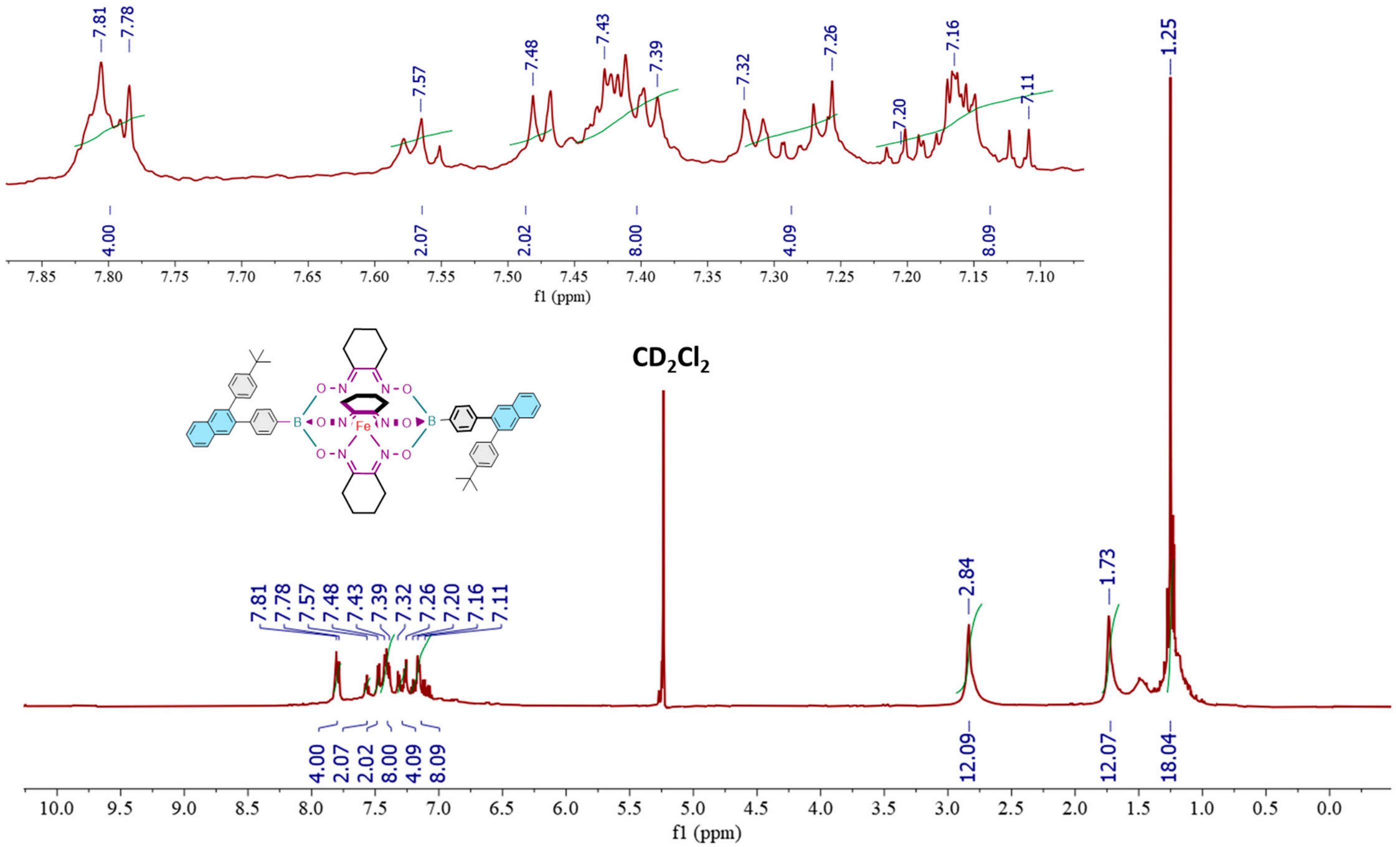

Figure S5: ¹H NMR spectrum of **CBM** (CD_2Cl_2, 600 MHz)

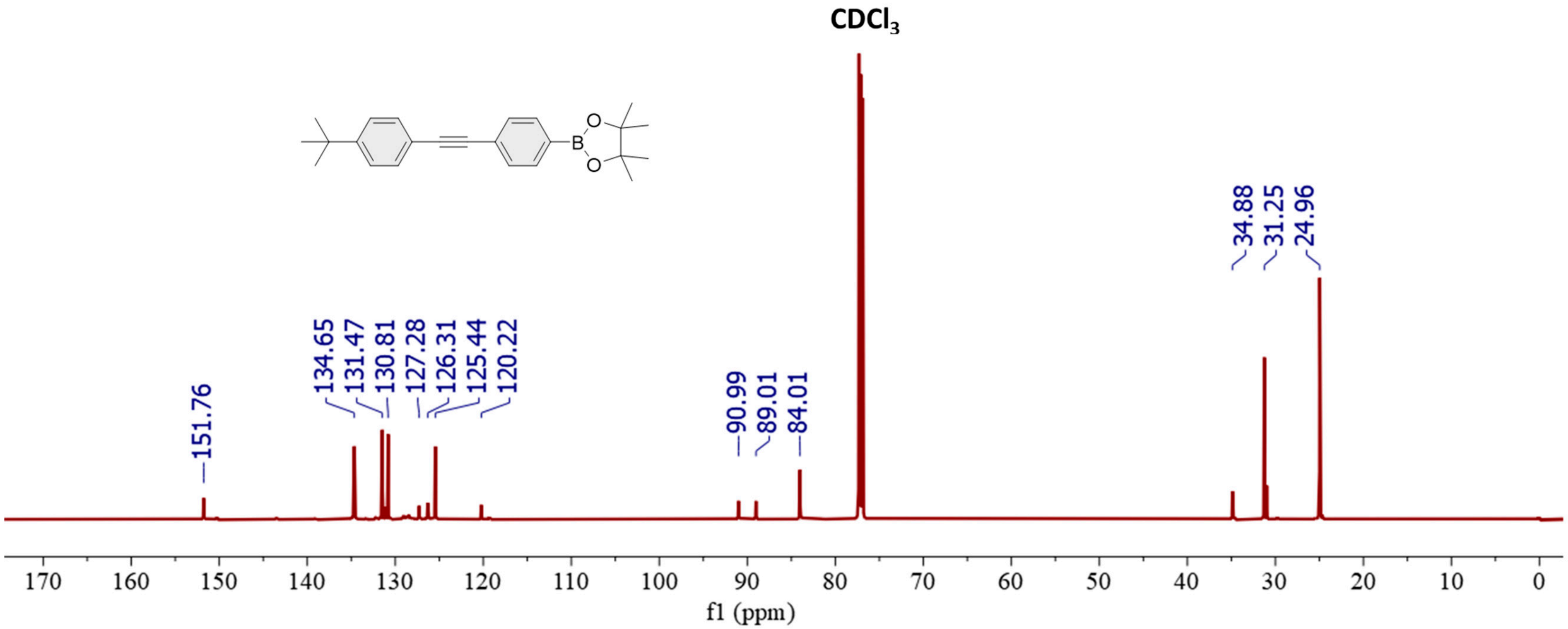

Figure S6: ^{13}C NMR spectrum of **4** ($CDCl_3$, 150 MHz)

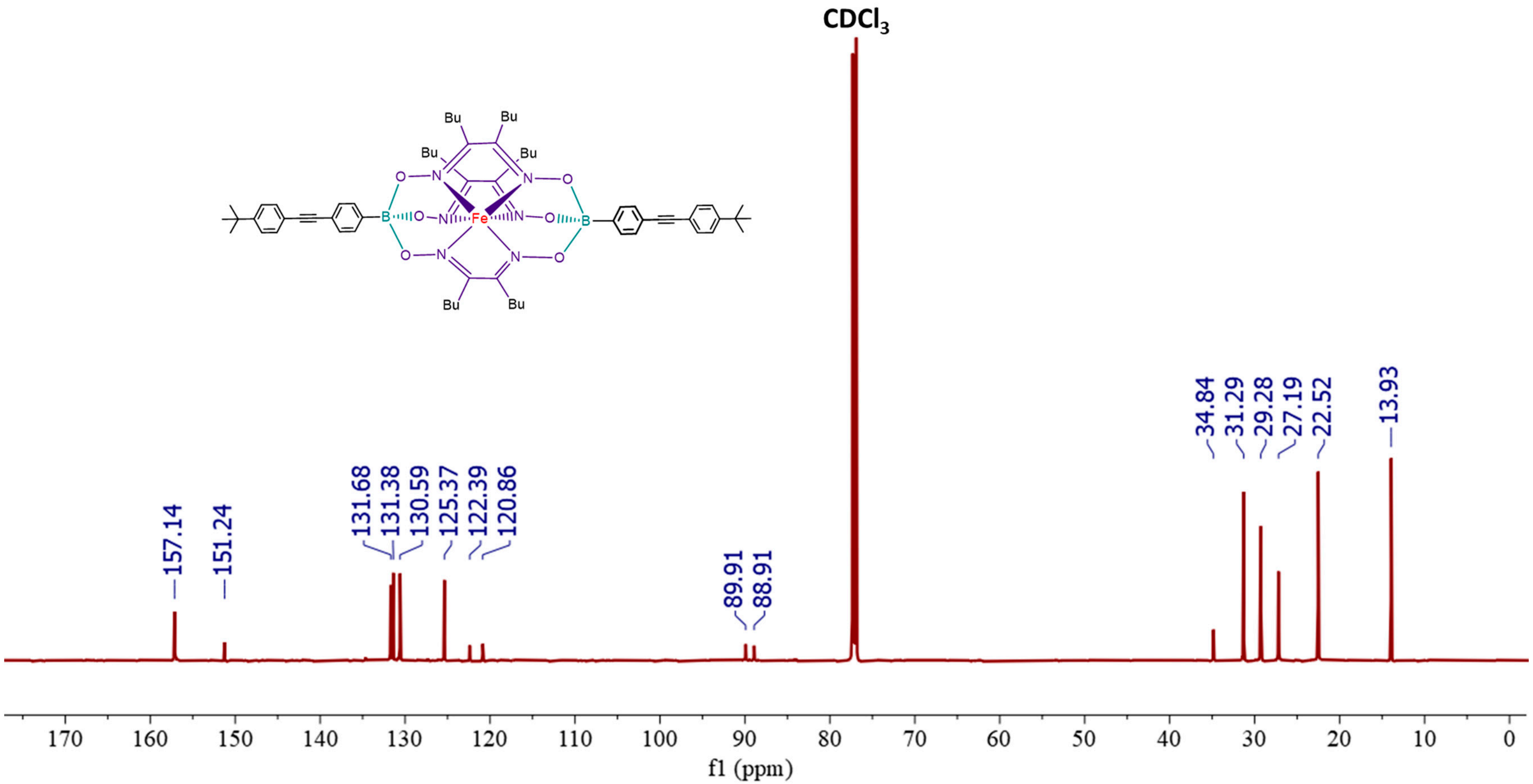

Figure S7: ^{13}C NMR spectrum of **CM1** ($CDCl_3$, 150 MHz)

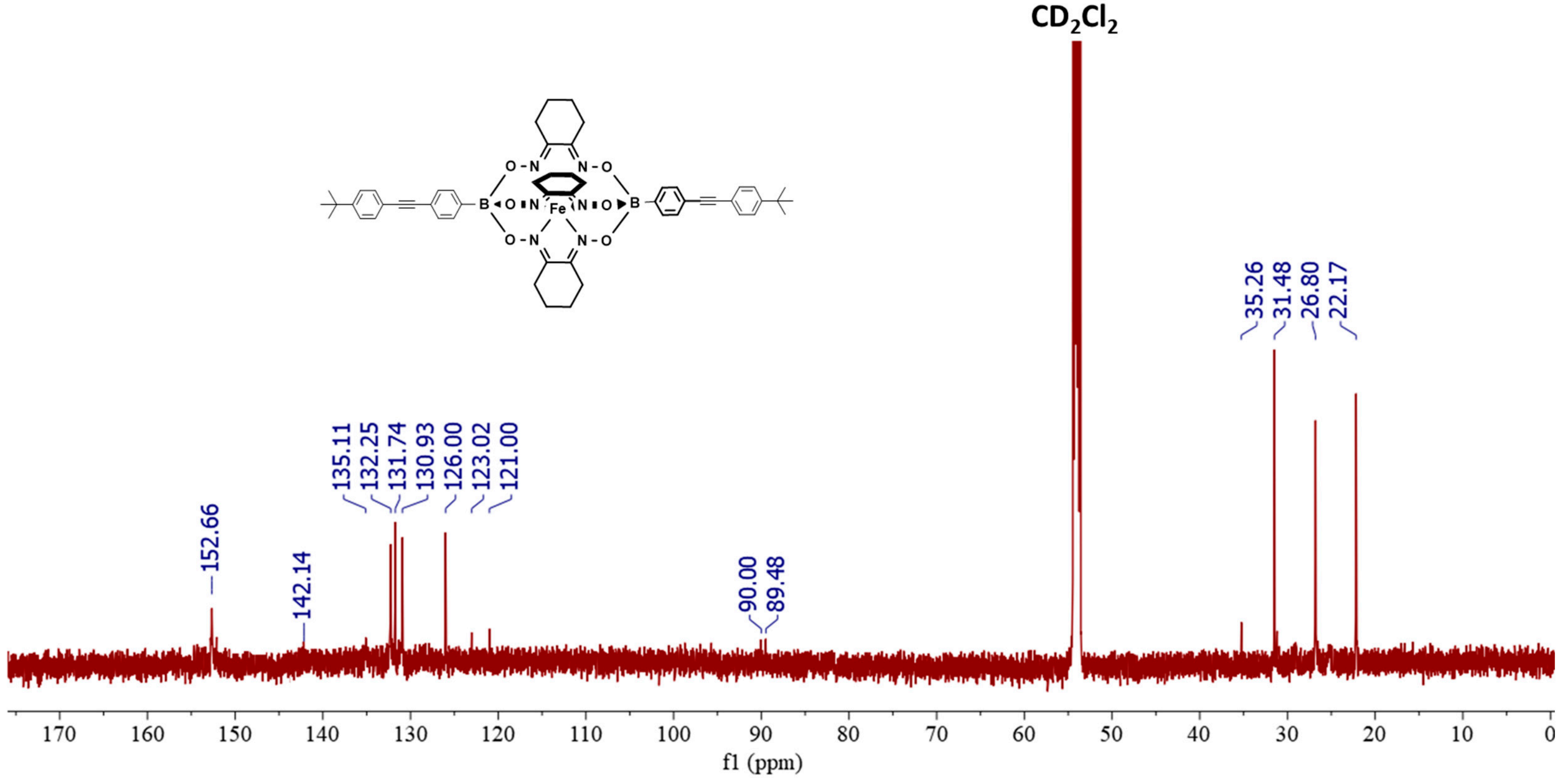

Figure S8: ^{13}C NMR spectrum of **CM2** (CD_2Cl_2, 150 MHz)

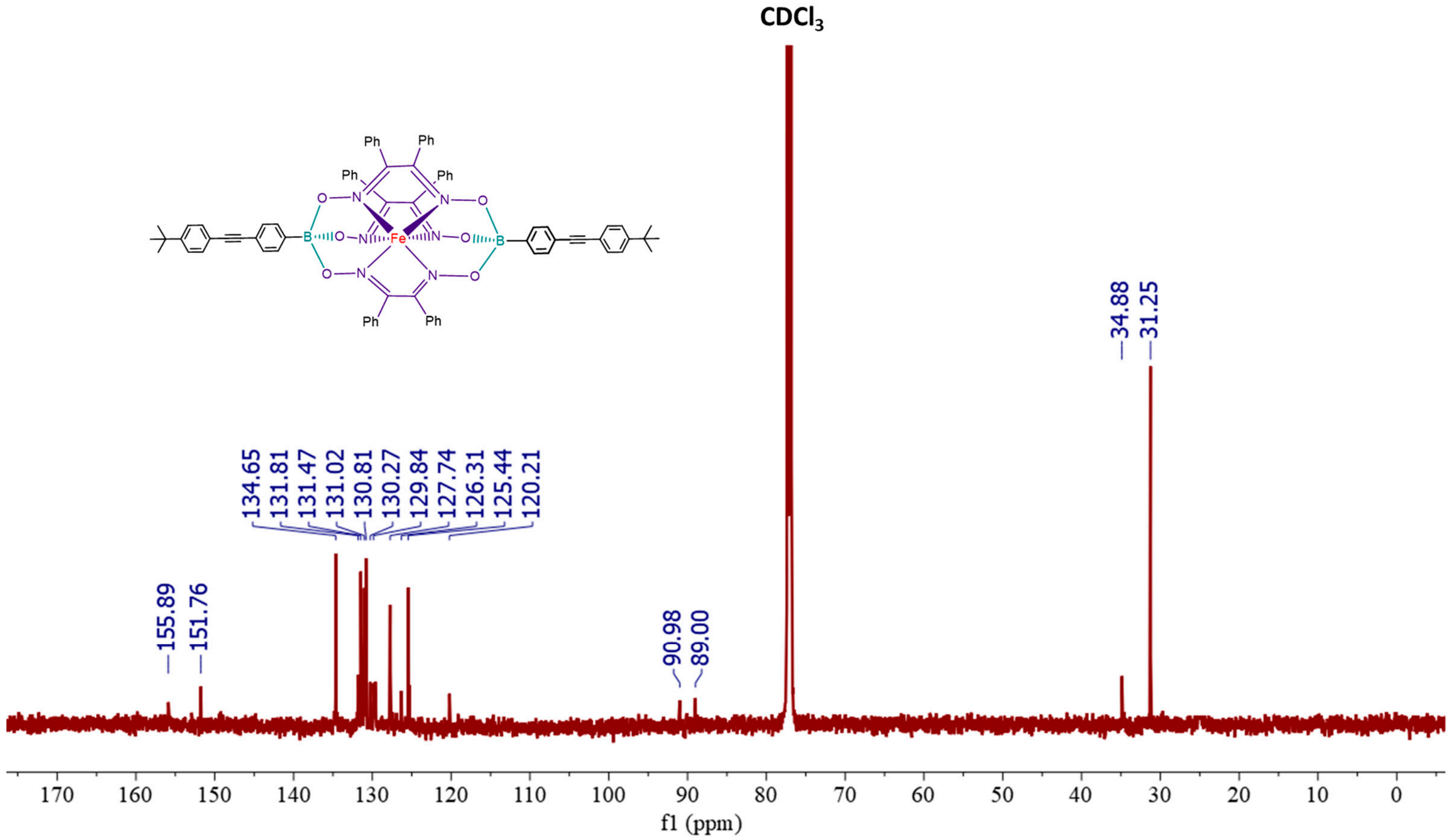

Figure S9: ^{13}C NMR spectrum of **CM3** ($CDCl_3$, 150 MHz)

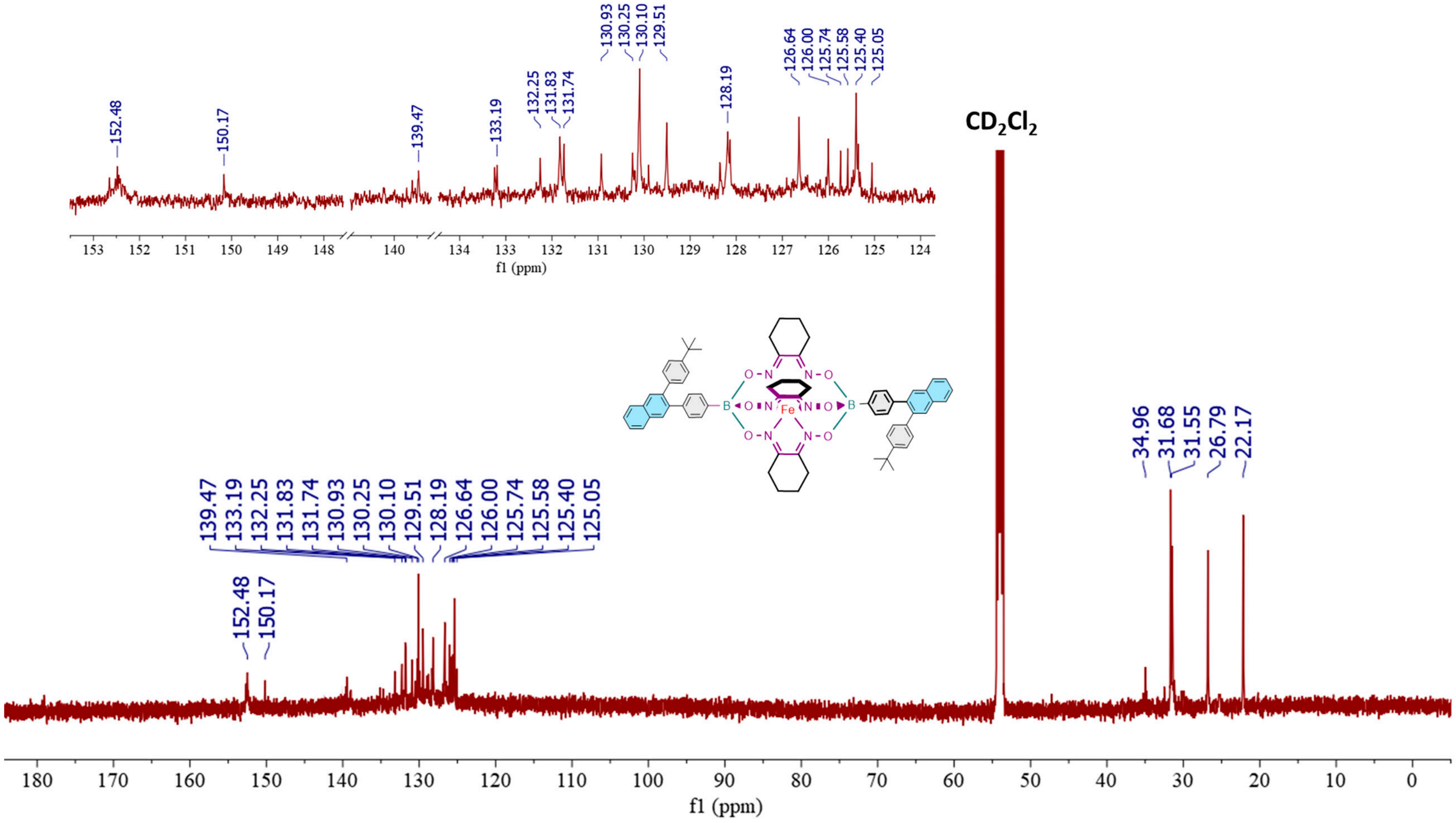

Figure S10: ^{13}C NMR spectrum of **CBM** (CD_2Cl_2, 150 MHz)

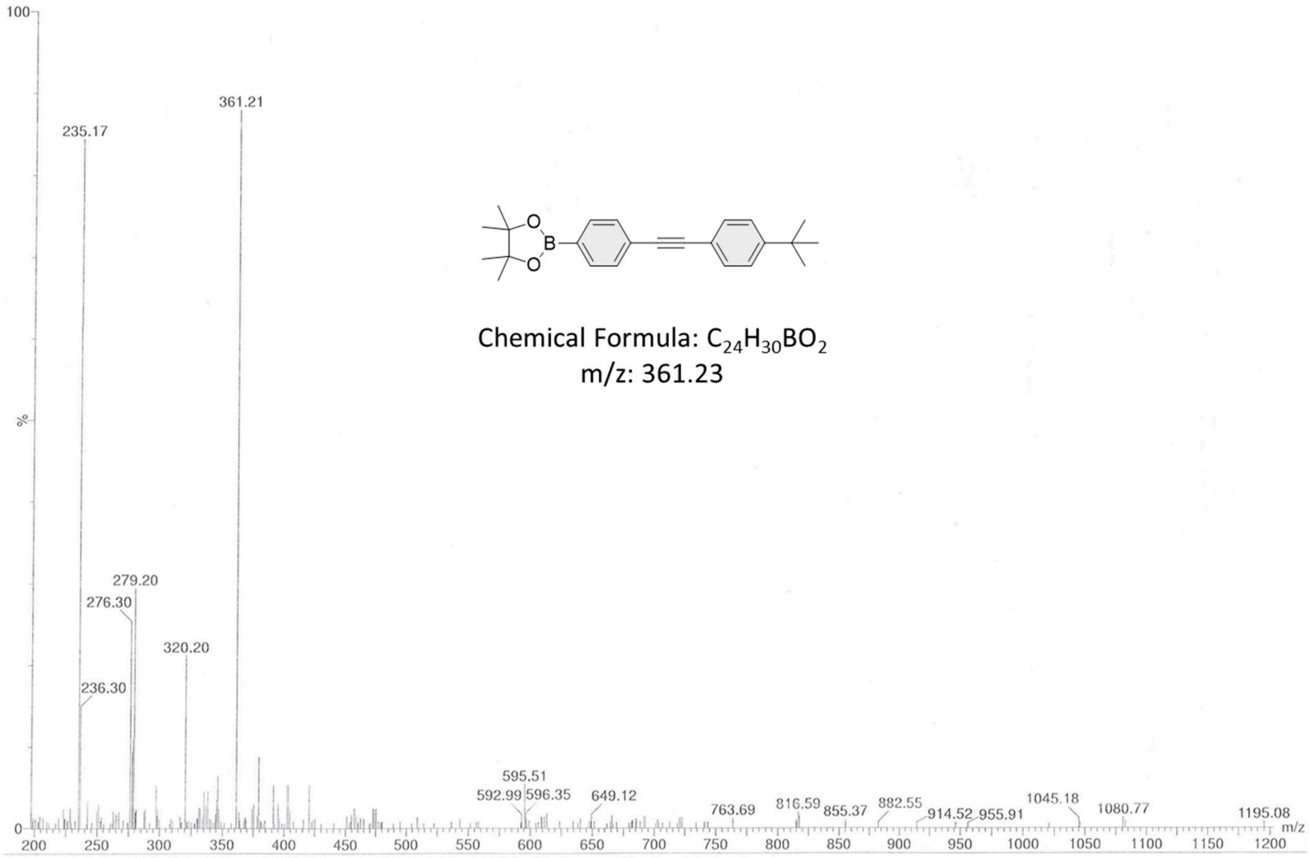

Figure S11: Electrospray ionization (ESI) mass spectrum of **4**

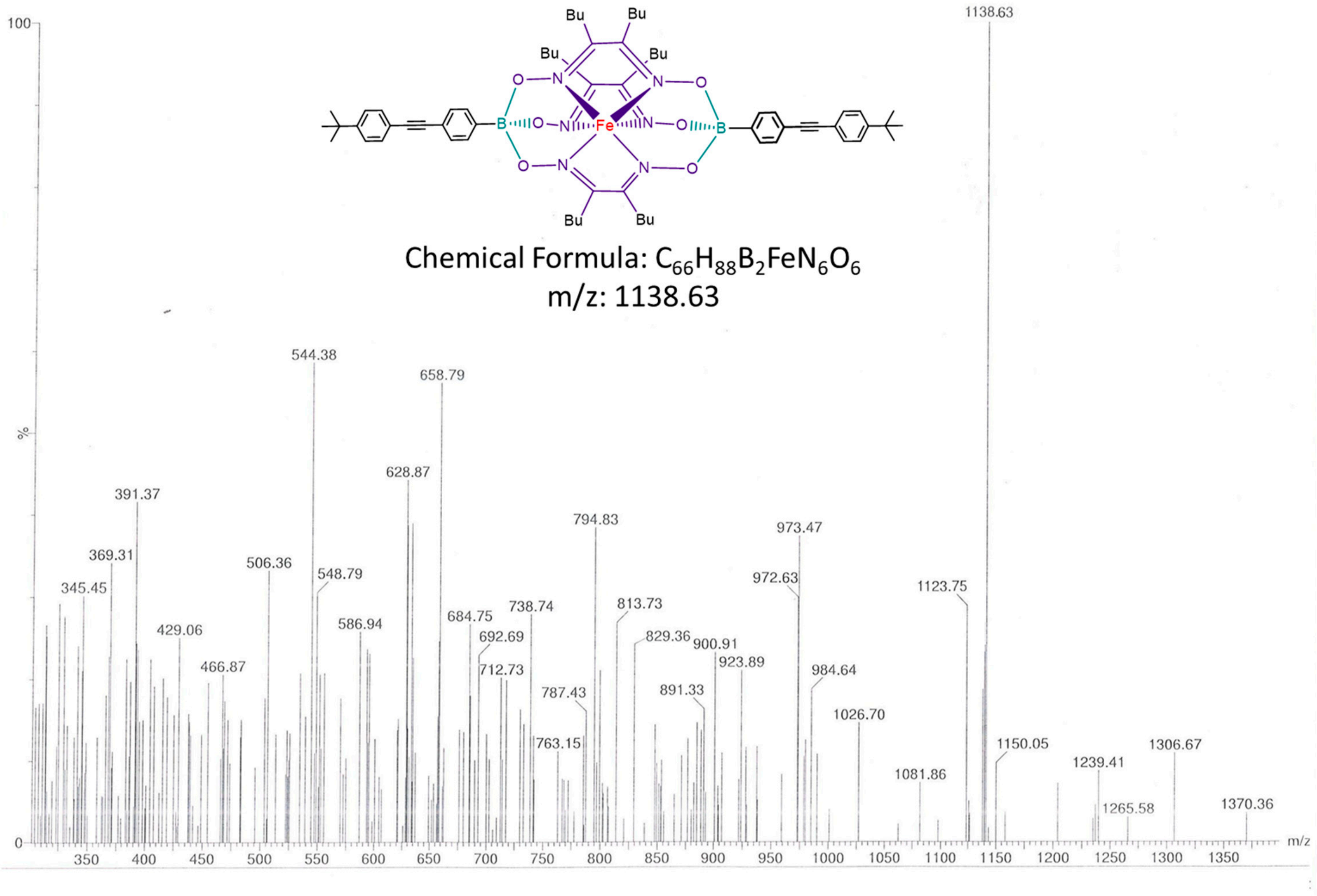

Figure S12: Electrospray ionization (ESI) mass spectrum of **CM1**

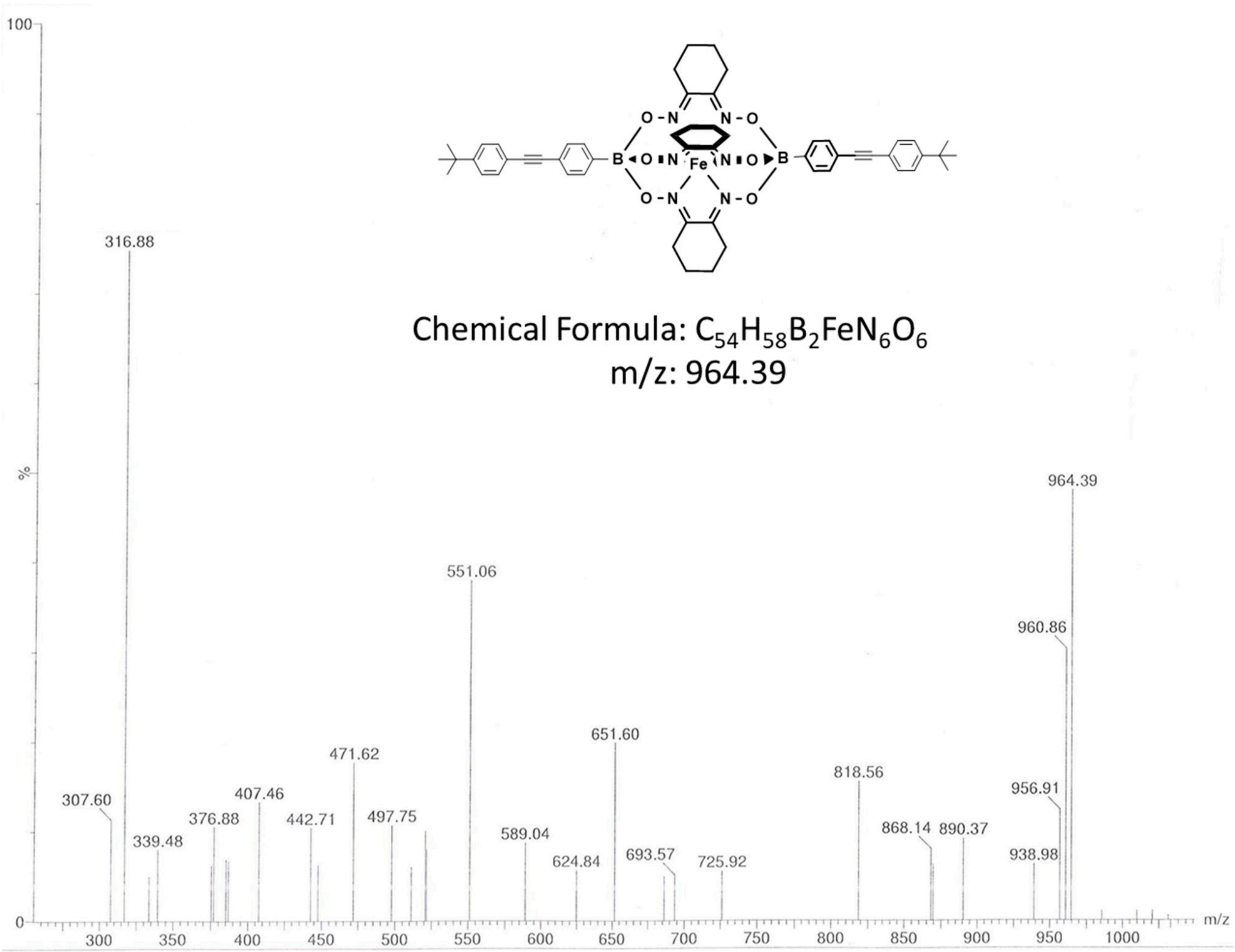

Figure S13: Electrospray ionization (ESI) mass spectrum of **CM2**

	C	**Δ**	H	**Δ**	N	**Δ**
Theoretical composition	74.42		5.12		6.68	
Elemental analysis 1	74.40	0.02	5.09	0.03	6.65	0.03
Elemental analysis 2	74.39	0.03	5.08	0.04	6.66	0.02
Elemental analysis 3	74.41	0.01	5.12	0.00	6.67	0.01
Average	74.40	0.02	5.10	0.02	6.66	0.02

Table S2: CHN Analysis of **CM3**

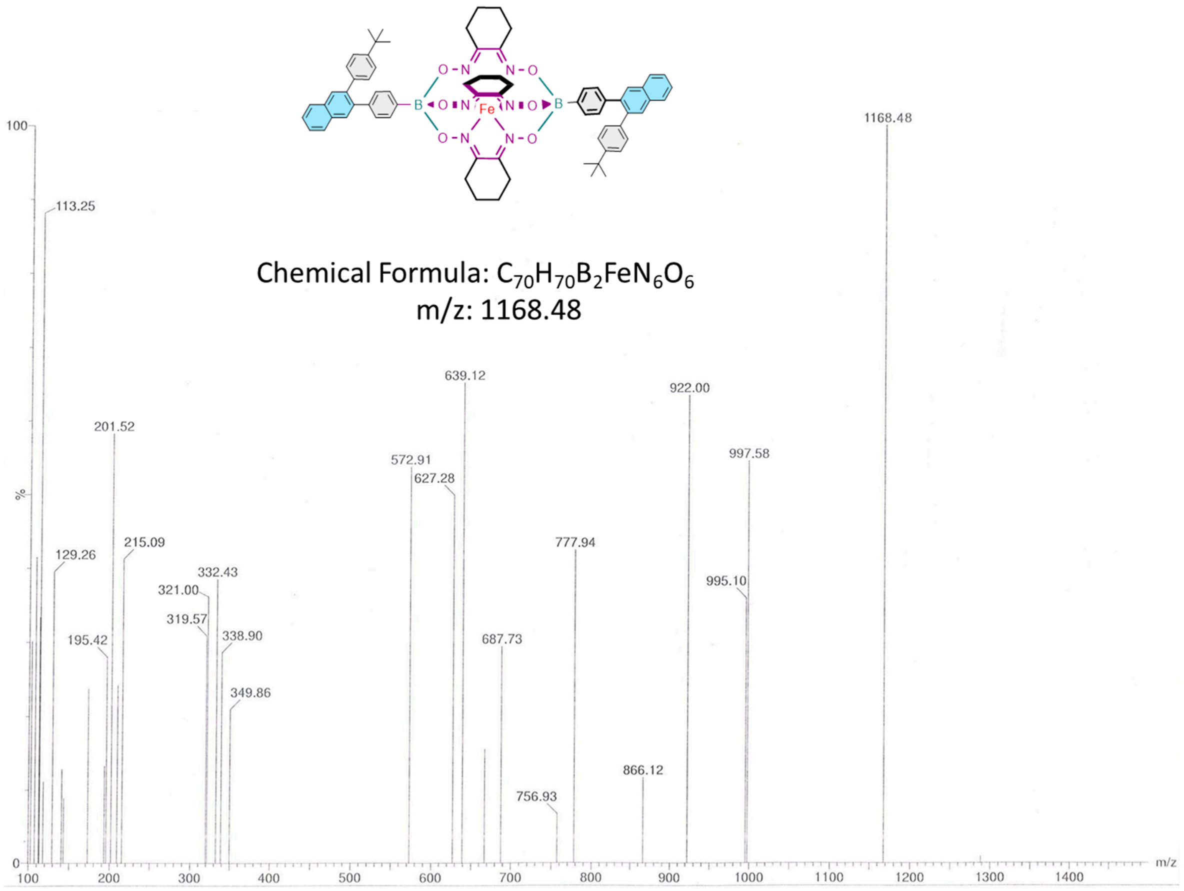

Figure S14: Electrospray ionization (ESI) mass spectrum of **CBM**

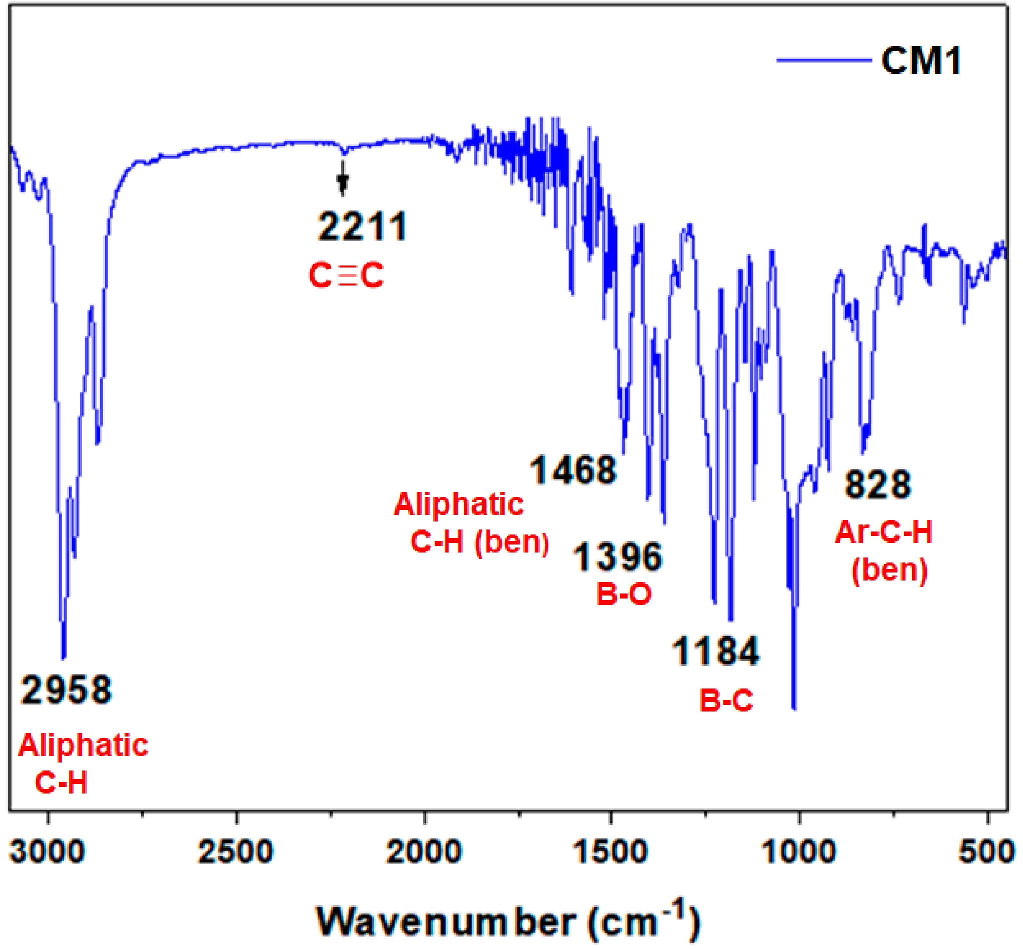

Figure S15: FT-IR spectrum of **CM1**

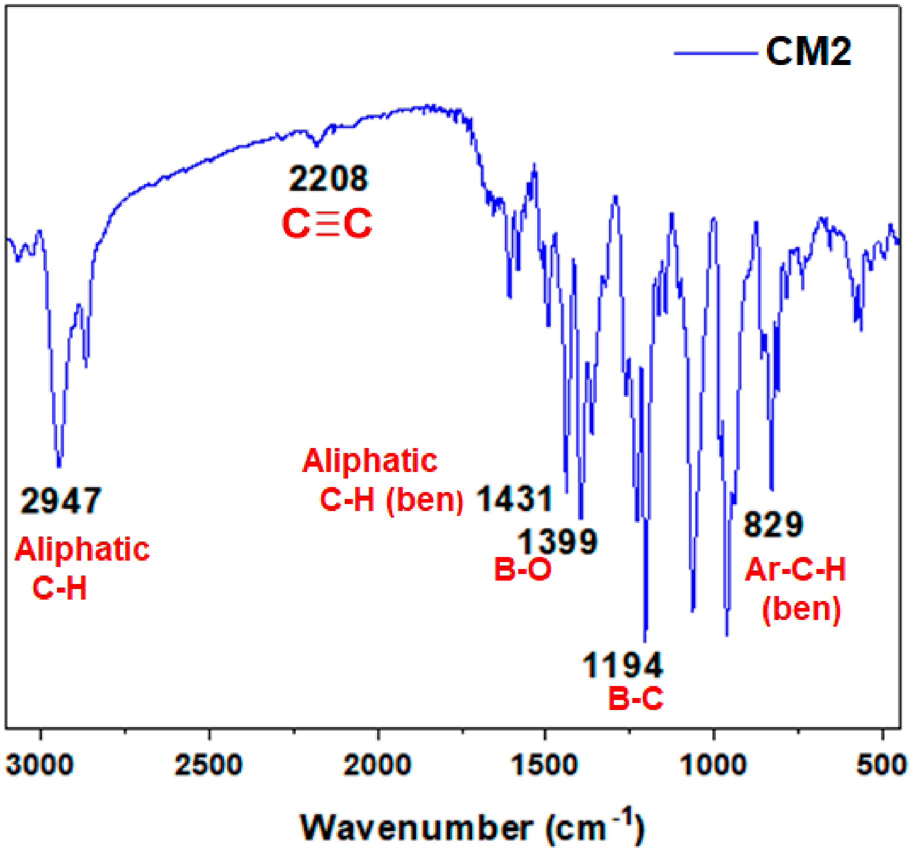

Figure S16: FT-IR spectrum of **CM2**

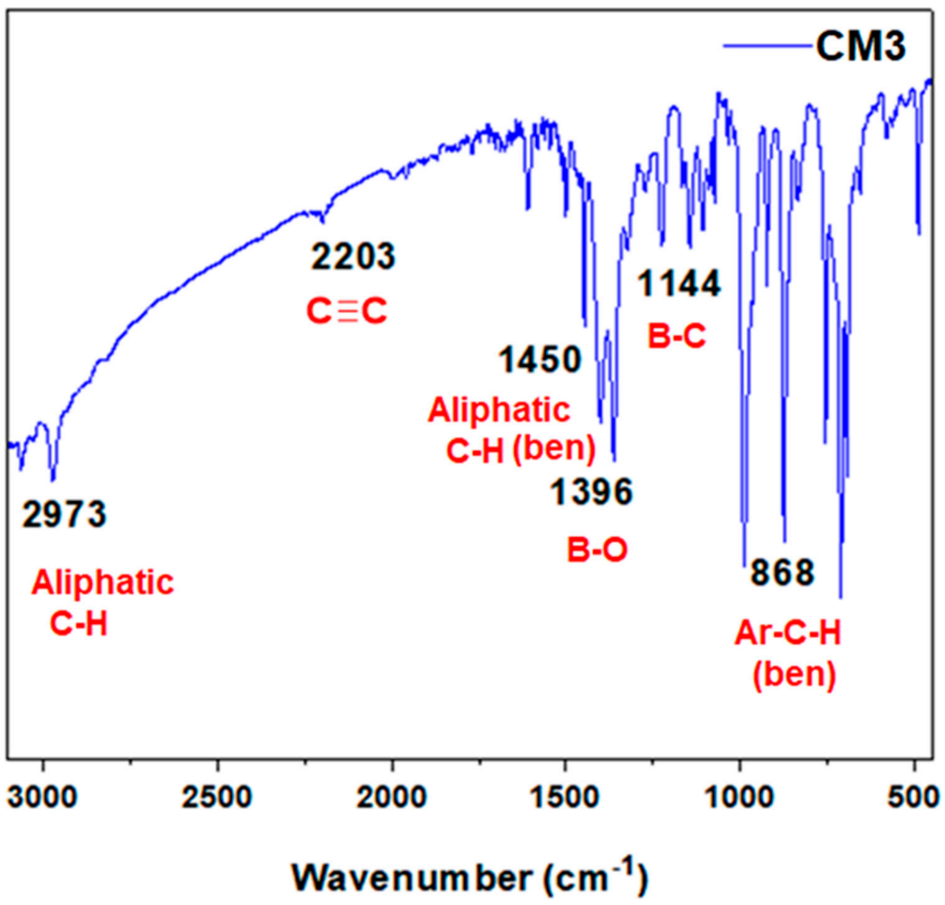

Figure S17: FT-IR spectrum of **CM3**

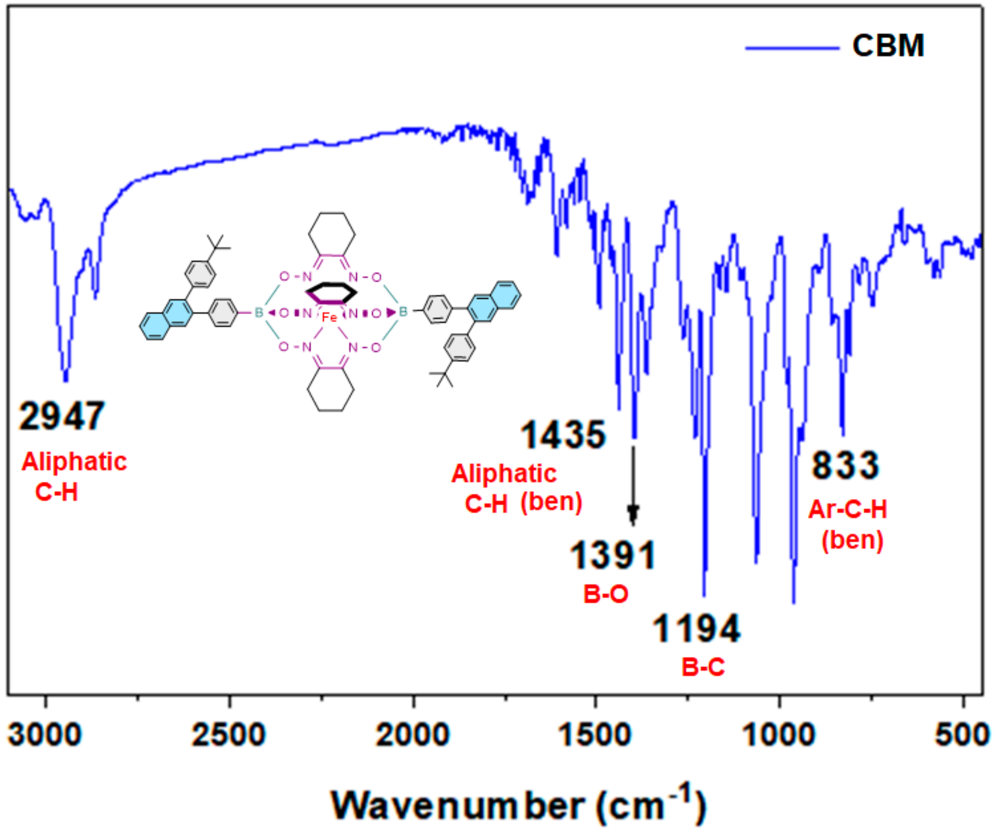

Figure S18: FT-IR spectrum of **CBM**

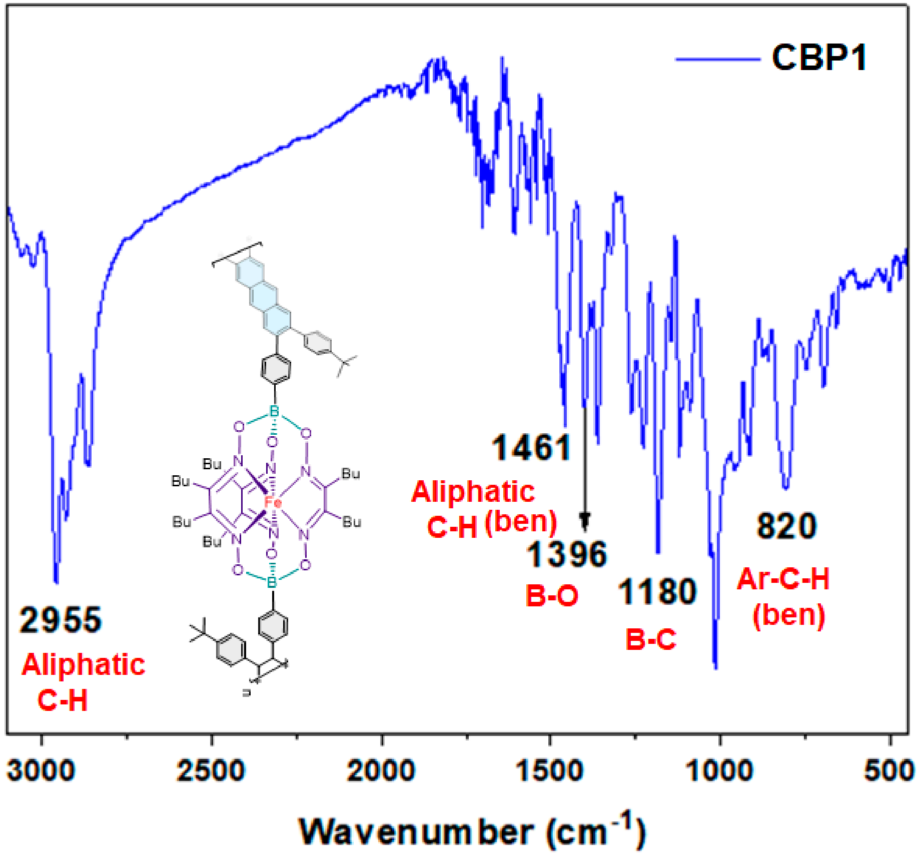

Figure S19: FT-IR spectrum of **CBP1**

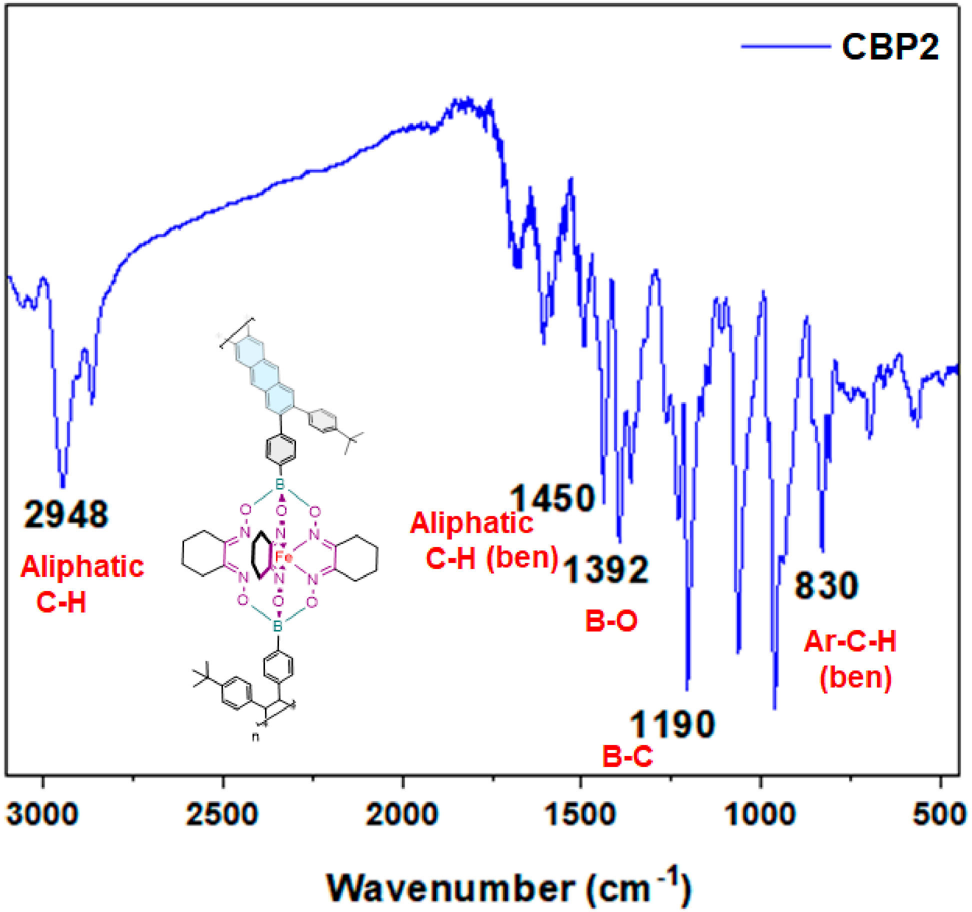

Figure S20: FT-IR spectrum of **CBP2**

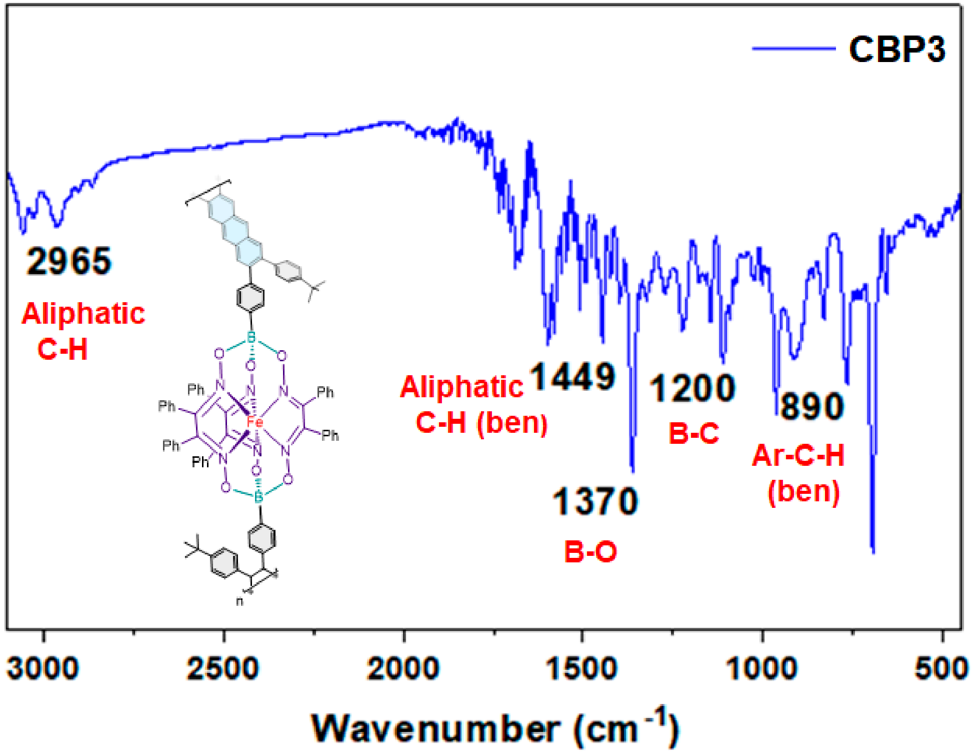

Figure S21: FT-IR spectrum of **CBP3**

CBP1	Peak BE
B 1s	191.15
C 1s	284.81
N 1s	400.54
O 1s	532.26
Fe 2p3	708.91
Fe2p1	721.99

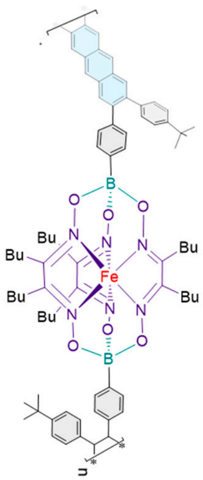

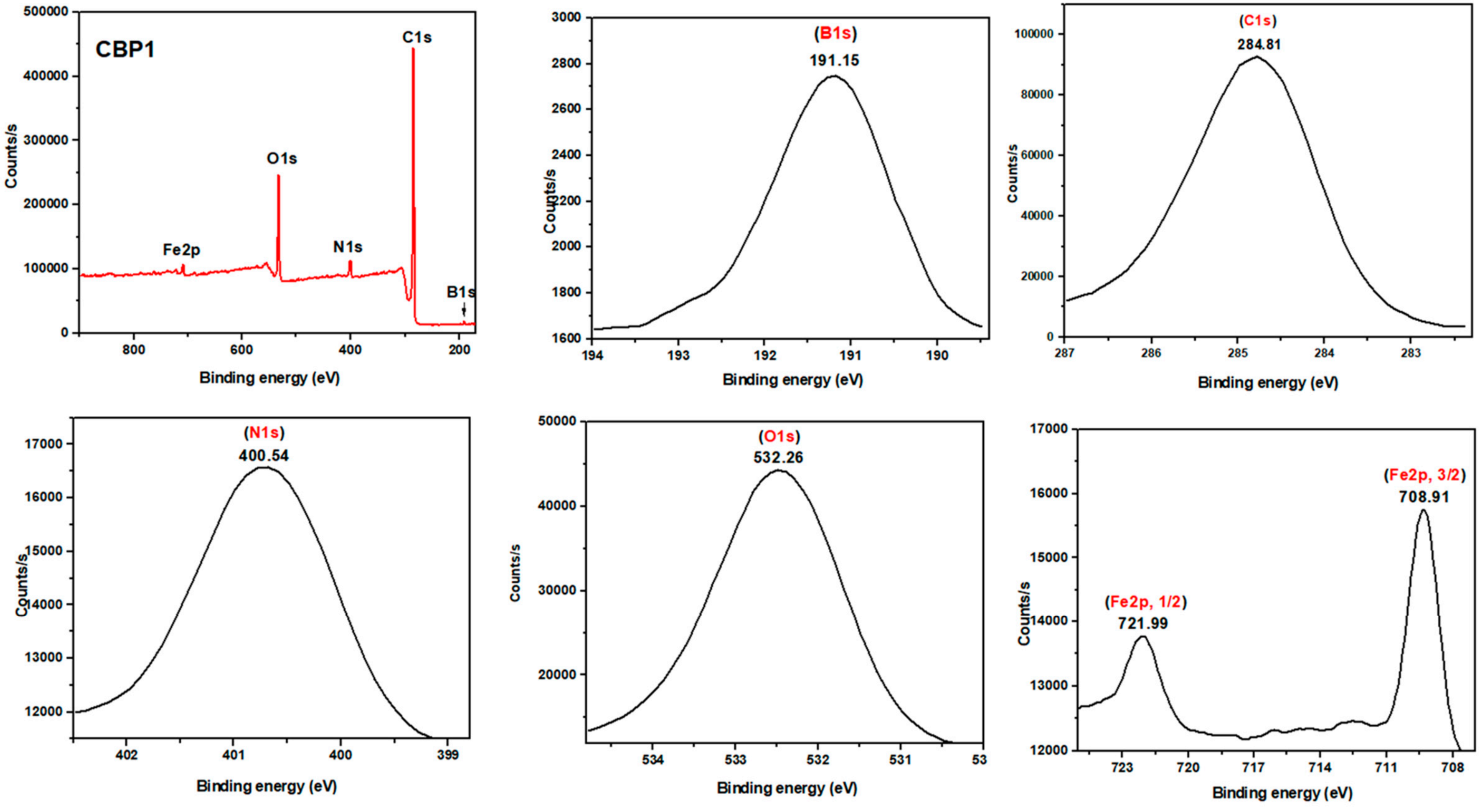

Figure S22: High-resolution XPS survey scan and spectra of B1s, C1s, N1s, O1s, and Fe2p of copolymer **CBP1**

CBP3	Peak BE
B 1s	191
C 1s	284.79
N 1s	400.19
O 1s	532.2
Fe 2p3	709.17
Fe 2p1	722.14

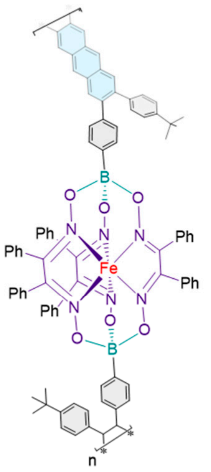

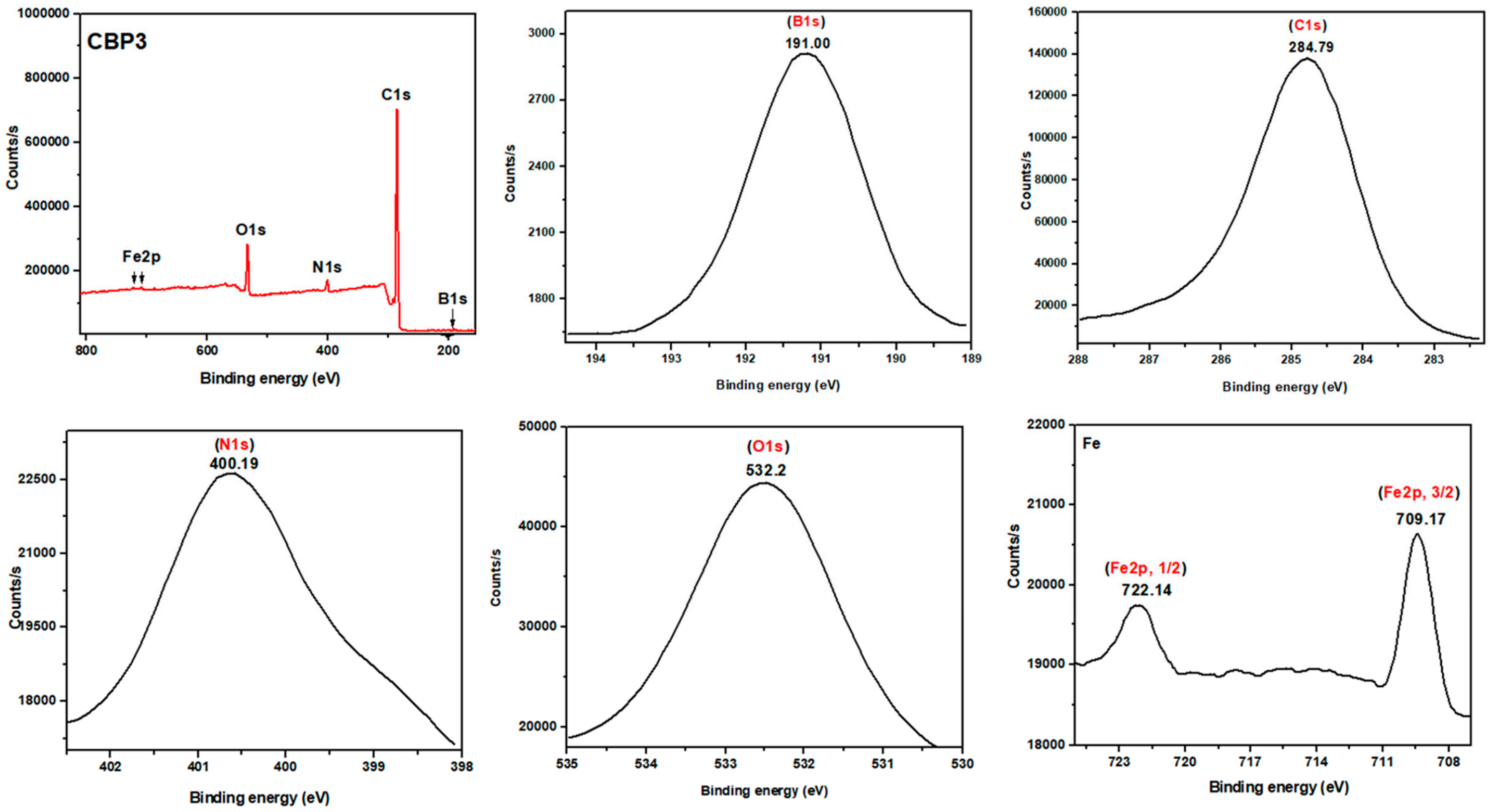

Figure S23: High-resolution XPS survey scan and spectra of B1s, C1s, N1s, O1s, and Fe2p of copolymer **CBP3**

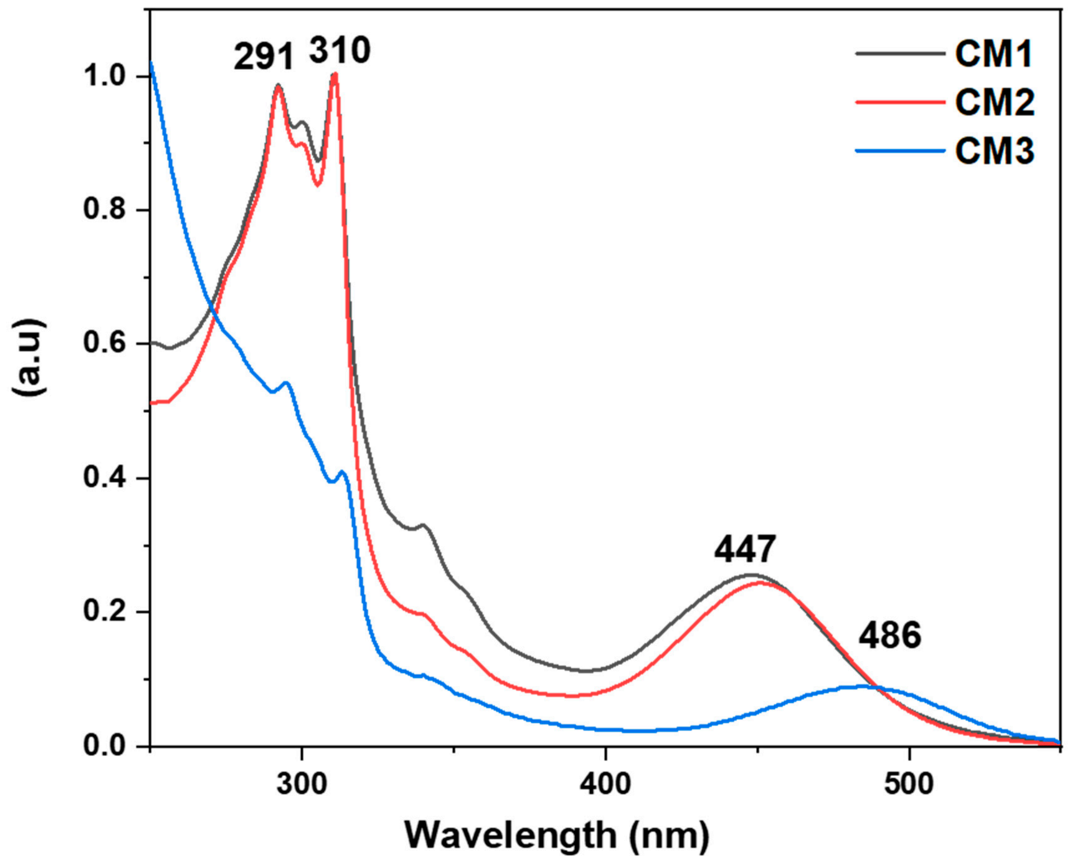

Figure S24: Normalized UV-VIS absorption spectra of **CM1-3** (CM = 10^{-8} M in THF)

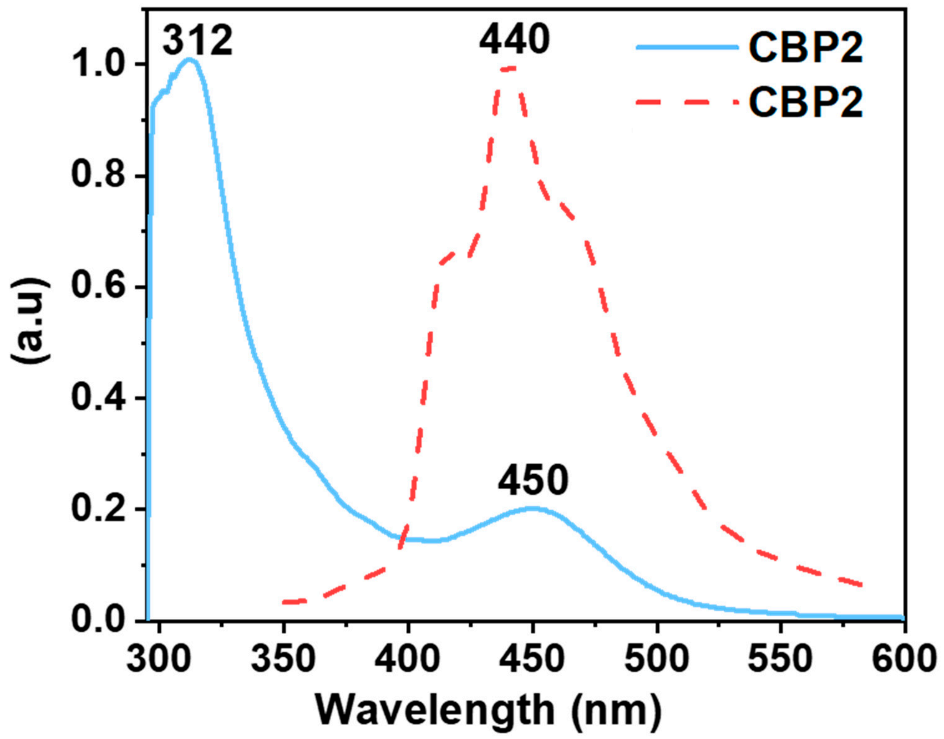

Figure S25: Normalized UV-VIS absorption (solid lines) and emission (dotted lines) spectra of **CBP2** (CM = 10^{-8} M in THF

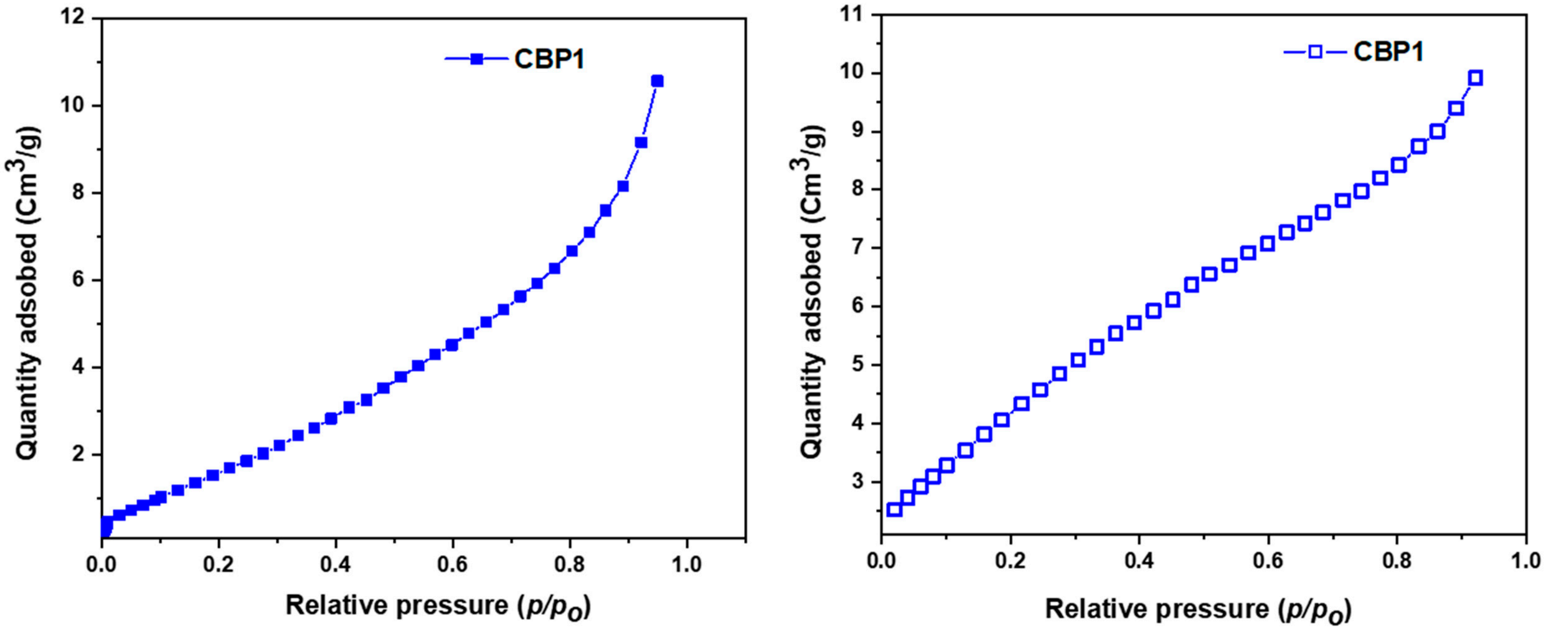

Figure S26: Nitrogen adsorption (left) and desorption isotherms (right) of **CBP1** measured at 77 K

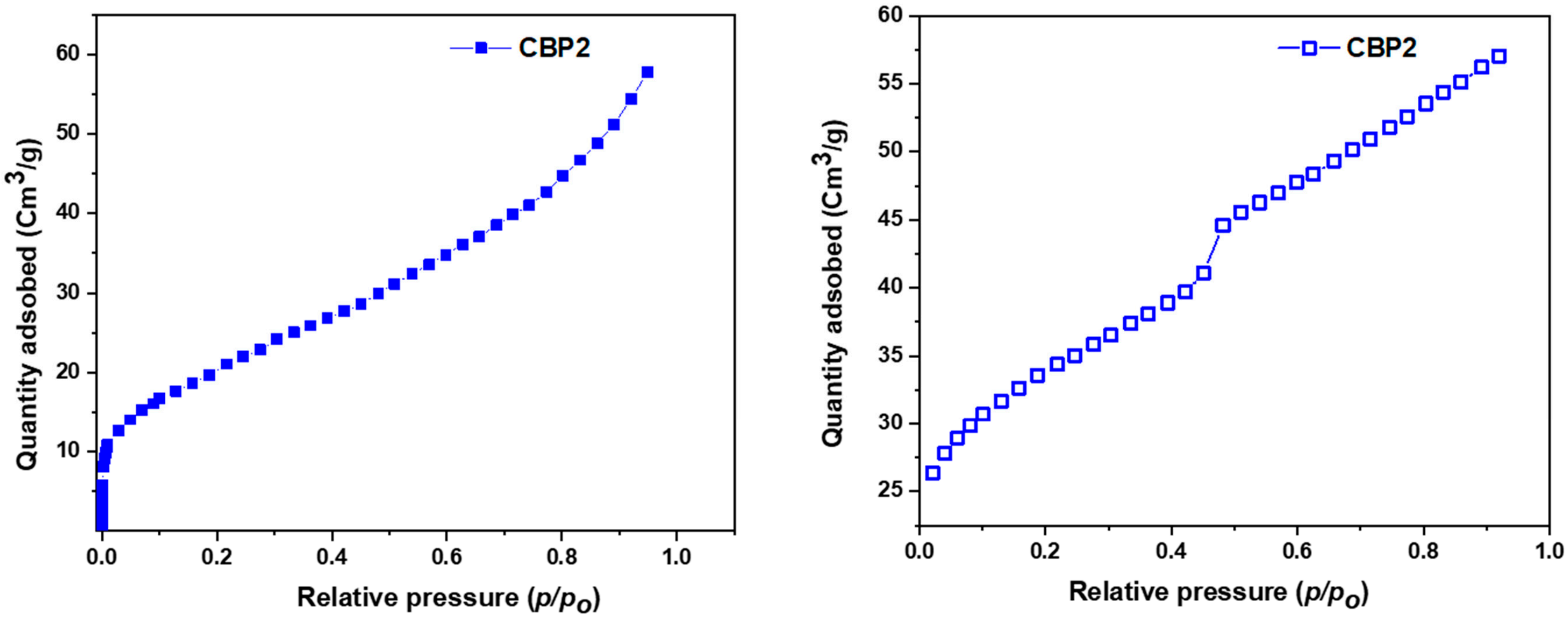

Figure S27: Nitrogen adsorption (left) and desorption isotherms (right) of **CBP2** measured at 77 K

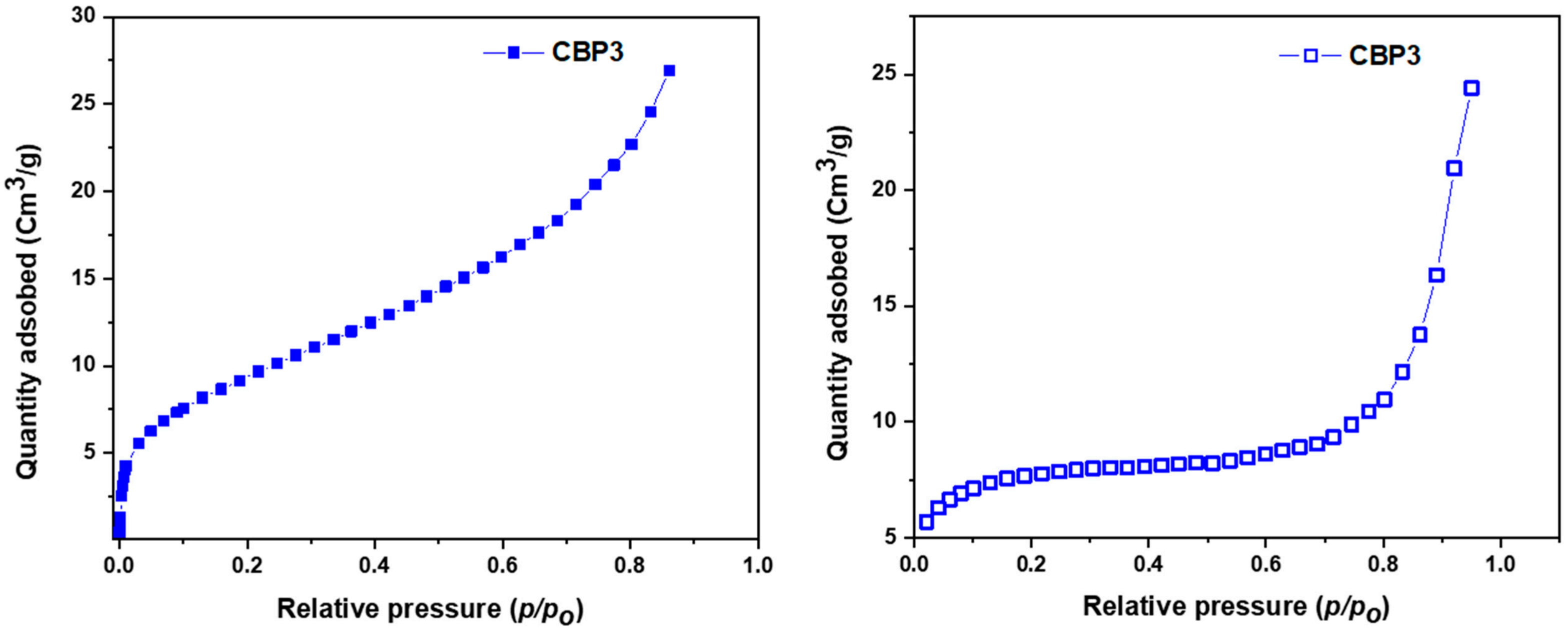

Figure S28: Nitrogen adsorption (left) and desorption isotherms (right) of **CBP3** measured at 77 K

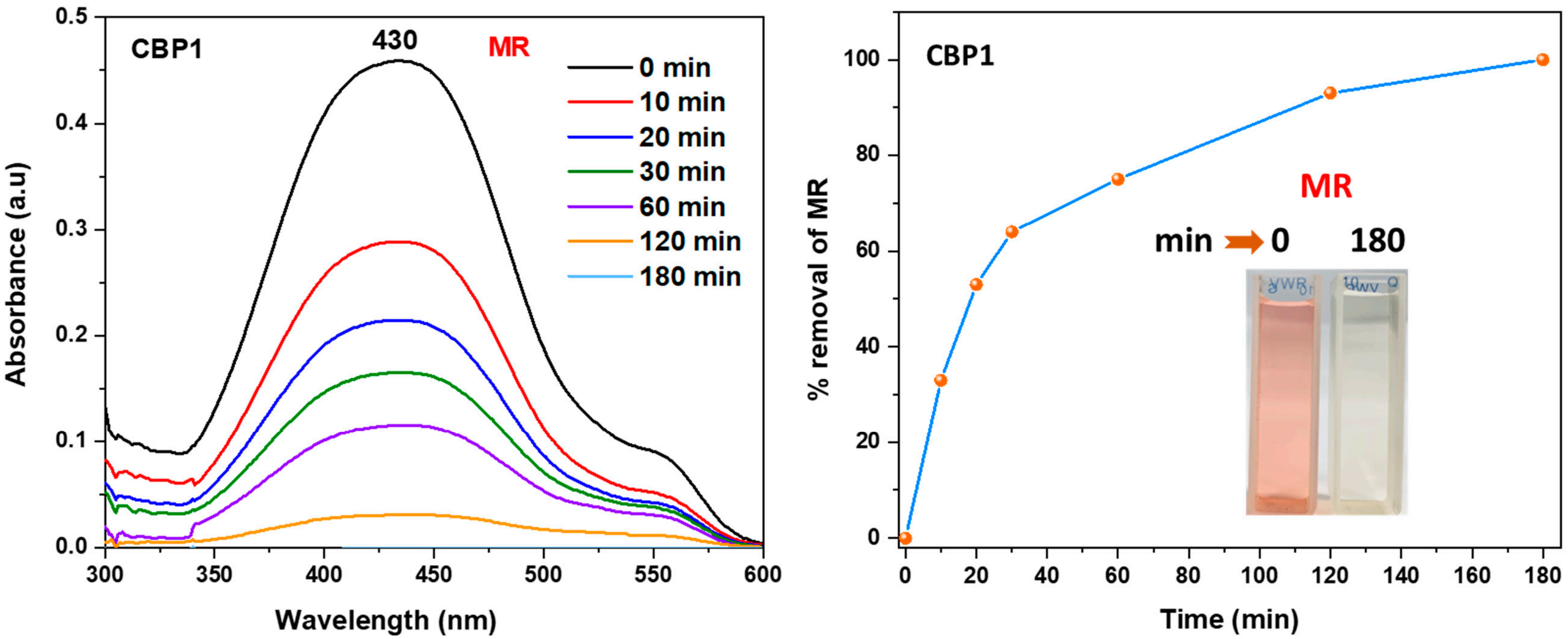

Figure S29: Methylene red (**MR**) dye adsorption by **CBP1** at various time intervals

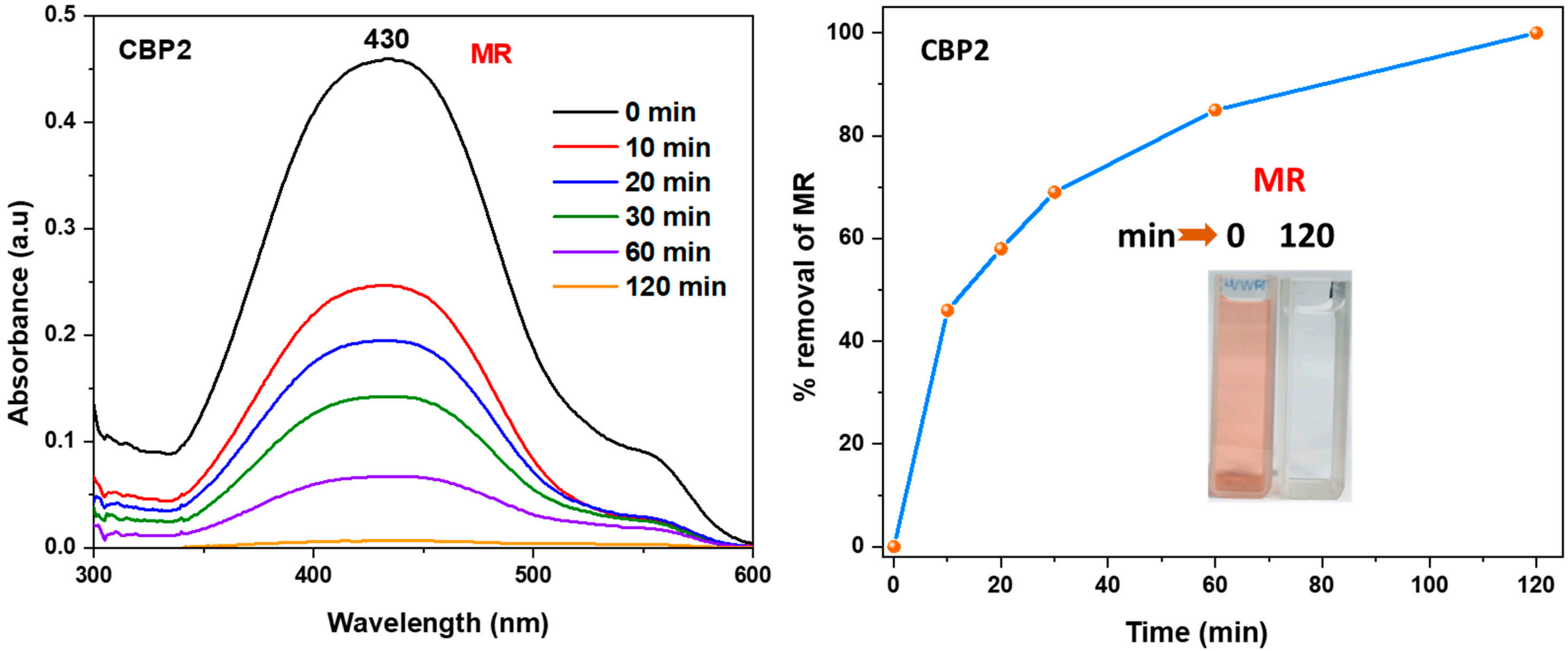

Figure S30: Methylene red (**MR**) dye adsorption by **CBP2** at various time intervals

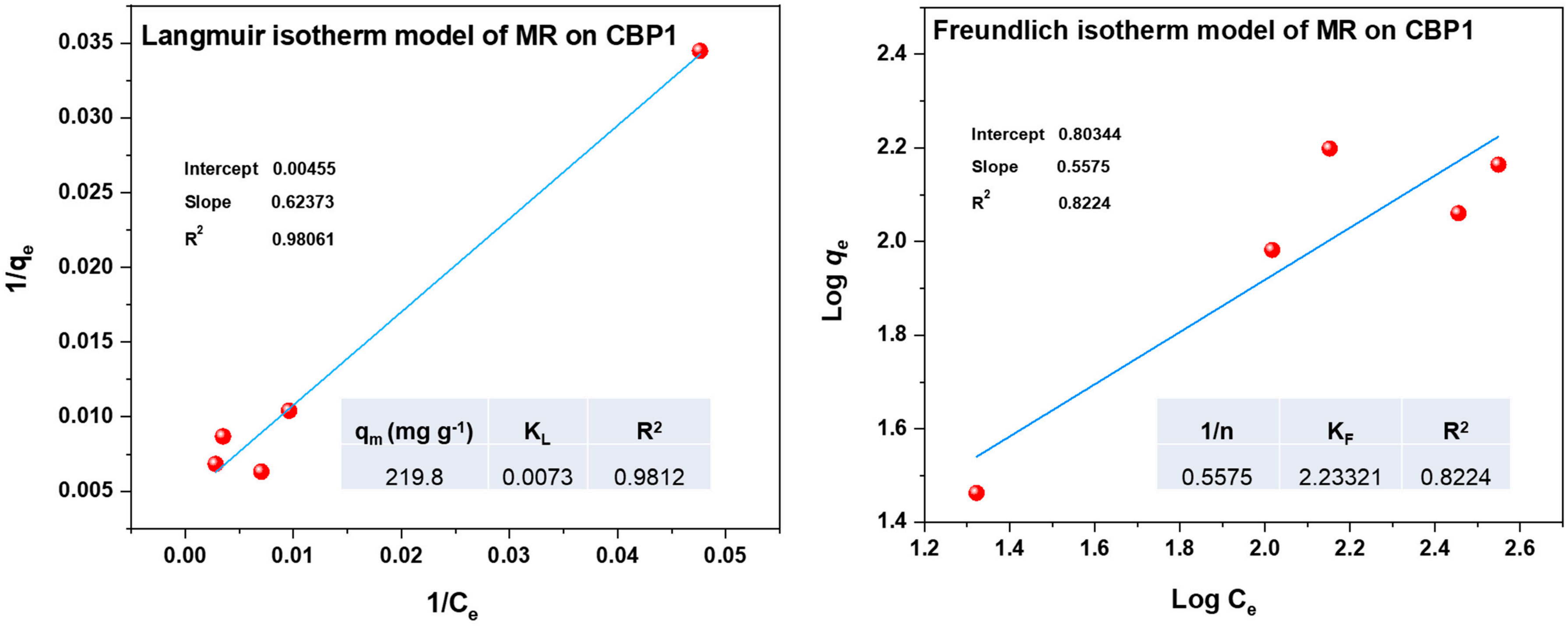

Figure S31: Linear Langmuir (left) and Freundlich (right) isotherm models of **MR** on **CBP1**

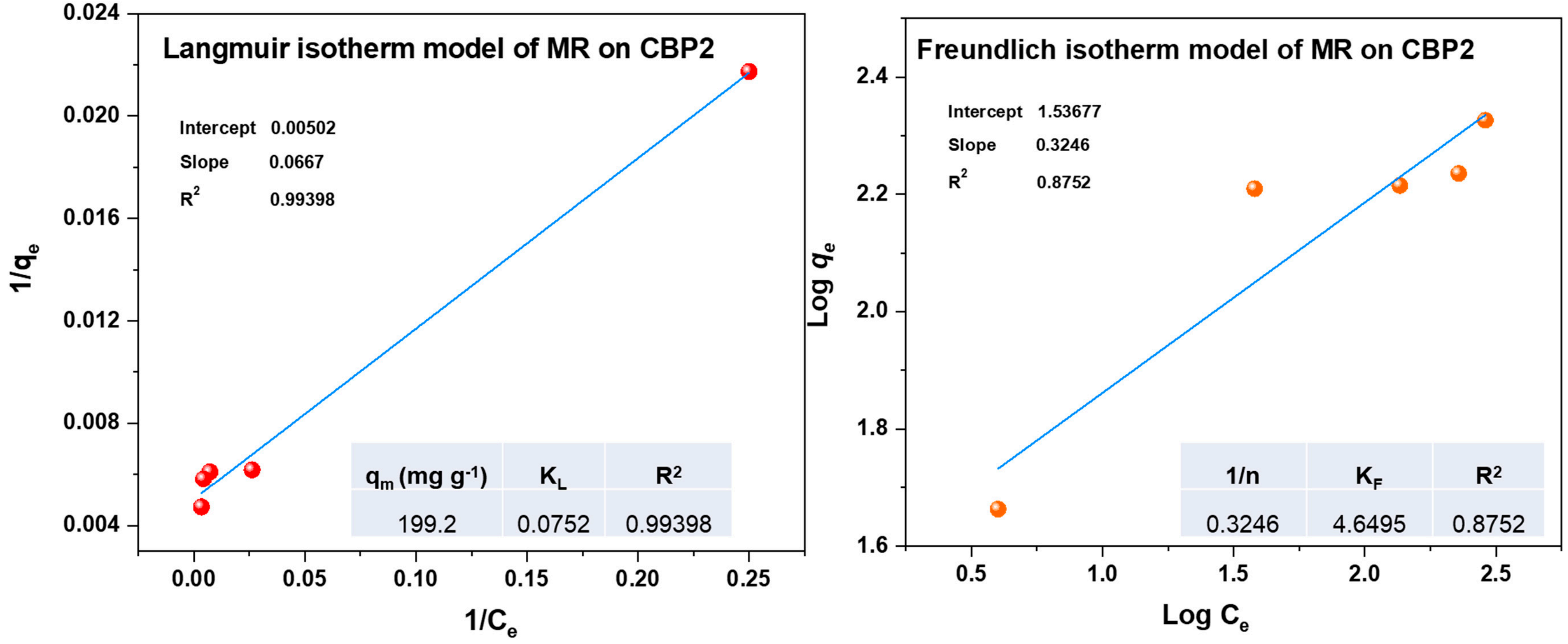

Figure S32: Linear Langmuir (left) and Freundlich (right) isotherm models of **MR** on **CBP2**

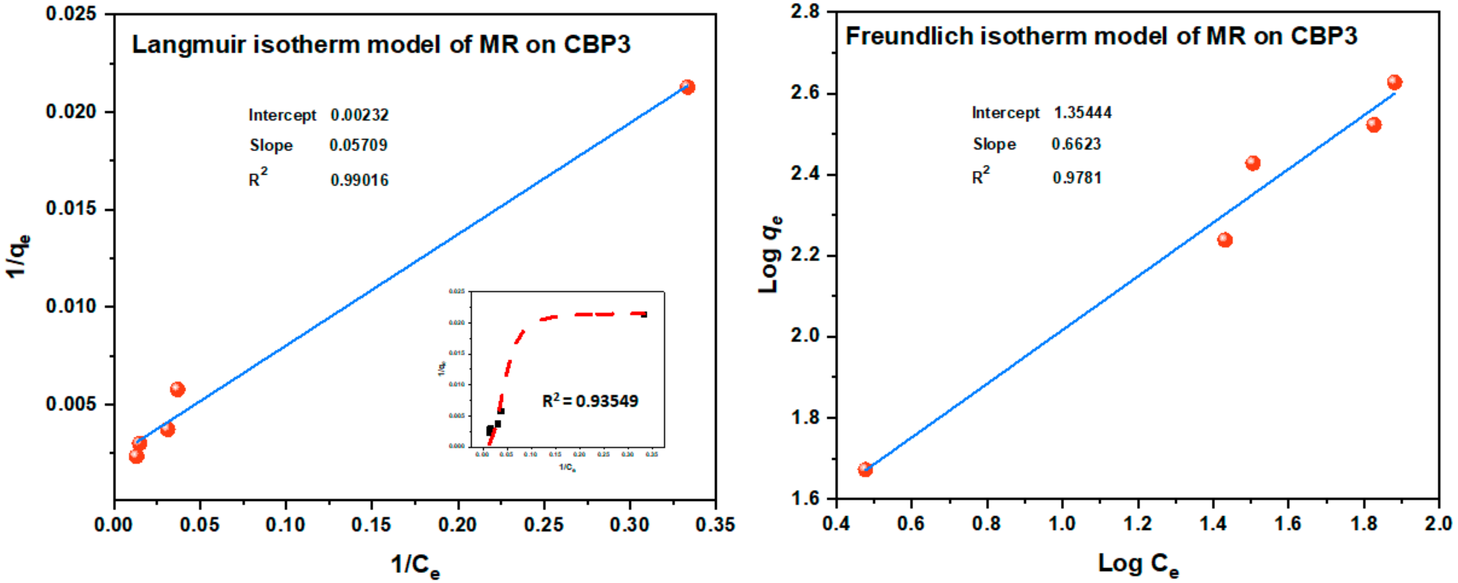

q_m (mg g^{-1})	K_L	R^2
431.03	0.0406	0.9901

1/n	K_F	R^2
0.6623	3.874591	0.9781

Figure S33: Linear Langmuir (left) and Freundlich (right) isotherm models of **MR** on **CBP3 (inset: non-linear Langmuir isotherm model of MR on CBP3)**

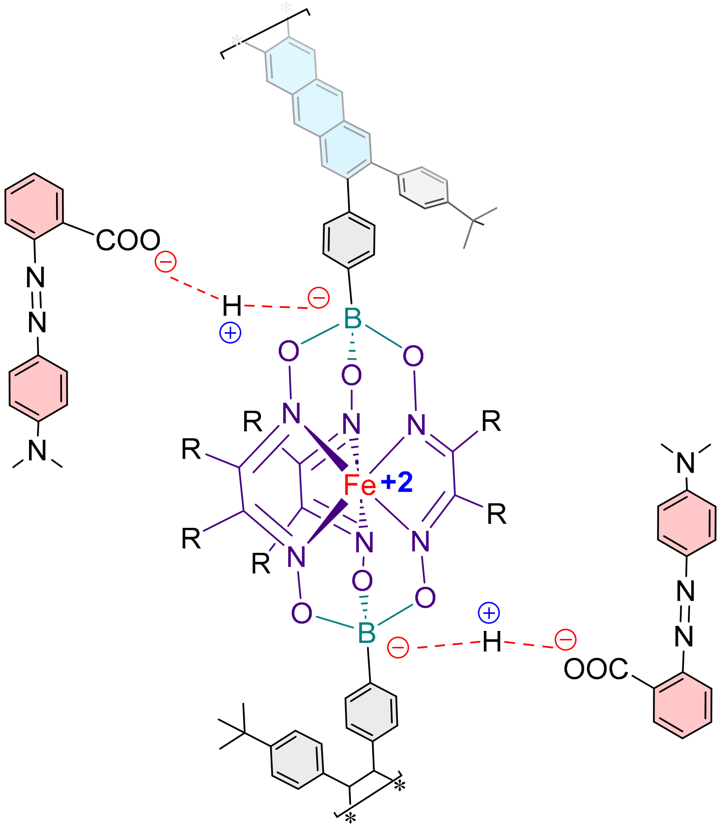

Figure S34: Plausible interaction of the copolymers and MR dye
